# Maternal Diet and the Serum Metabolome in Pregnancy: Robust Dietary Biomarkers Generalizable to a Multiethnic Birth Cohort

**DOI:** 10.1093/cdn/nzaa144

**Published:** 2020-09-02

**Authors:** Russell J de Souza, Meera Shanmuganathan, Amel Lamri, Stephanie A Atkinson, Allan Becker, Dipika Desai, Milan Gupta, Piush J Mandhane, Theo J Moraes, Katherine M Morrison, Padmaja Subbarao, Koon K Teo, Stuart E Turvey, Natalie C Williams, Philip Britz-McKibbin, Sonia S Anand

**Affiliations:** Department of Health Research Methods, Evidence, and Impact, Faculty of Health Sciences, McMaster University, Hamilton, ON, Canada; Centre for Metabolism, Obesity and Diabetes Research, McMaster University, Hamilton, ON, Canada; Population Health Research Institute, Hamilton, ON, Canada; Department of Chemistry and Chemical Biology, McMaster University, Hamilton, ON, Canada; Population Health Research Institute, Hamilton, ON, Canada; Department of Medicine, McMaster University, Hamilton, ON, Canada; Department of Pediatrics, McMaster University, Hamilton, ON, Canada; Children's Hospital Research Institute, University of Manitoba, Winnipeg, MB, Canada; Department of Health Research Methods, Evidence, and Impact, Faculty of Health Sciences, McMaster University, Hamilton, ON, Canada; Population Health Research Institute, Hamilton, ON, Canada; Department of Medicine, McMaster University, Hamilton, ON, Canada; Canadian Collaborative Research Network, Brampton, ON, Canada; Department of Pediatrics, University of Alberta, Edmonton, AB, Canada; Hospital for Sick Children, Toronto, ON, Canada; Centre for Metabolism, Obesity and Diabetes Research, McMaster University, Hamilton, ON, Canada; Population Health Research Institute, Hamilton, ON, Canada; Department of Pediatrics, McMaster University, Hamilton, ON, Canada; Hospital for Sick Children, Toronto, ON, Canada; University of Toronto, Toronto, ON, Canada; Population Health Research Institute, Hamilton, ON, Canada; Department of Medicine, McMaster University, Hamilton, ON, Canada; BC Children's Hospital and The University of British Columbia, Vancouver, BC, Canada; Department of Medicine, McMaster University, Hamilton, ON, Canada; Department of Chemistry and Chemical Biology, McMaster University, Hamilton, ON, Canada; Department of Health Research Methods, Evidence, and Impact, Faculty of Health Sciences, McMaster University, Hamilton, ON, Canada; Centre for Metabolism, Obesity and Diabetes Research, McMaster University, Hamilton, ON, Canada; Population Health Research Institute, Hamilton, ON, Canada; Department of Medicine, McMaster University, Hamilton, ON, Canada

**Keywords:** diet, metabolomics, pregnancy, dietary biomarkers, serum, birth cohort

## Abstract

**Background:**

Advances in metabolomics are anticipated to decipher associations between dietary exposures and health. Replication biomarker studies in different populations are critical to demonstrate generalizability.

**Objectives:**

To identify and validate robust serum metabolites associated with diet quality and specific foods in a multiethnic cohort of pregnant women.

**Design:**

In this cross-sectional analysis of 3 multiethnic Canadian birth cohorts, we collected semiquantitative FFQ and serum data from 900 women at the second trimester of pregnancy. We calculated a diet quality score (DQS), defined as daily servings of “healthy” minus “unhealthy” foods. Serum metabolomics was performed by multisegment injection-capillary electrophoresis-mass spectrometry, and specific serum metabolites associated with maternal DQSs were identified. We combined the results across all 3 cohorts using meta-analysis to classify robust dietary biomarkers (*r* > ± 0.1; *P* < 0.05).

**Results:**

Diet quality was higher in the South Asian birth cohort (mean DQS = 7.1) than the 2 white Caucasian birth cohorts (mean DQS <3.2). Sixty-six metabolites were detected with high frequency (>75%) and adequate precision (CV <30%), and 47 were common to all cohorts. Hippuric acid was positively associated with healthy diet score in all cohorts, and with the overall DQS only in the primarily white Caucasian cohorts. We observed robust correlations between: *1*) proline betaine—citrus foods; *2*) 3-methylhistidine—red meat, chicken, and eggs; *3*) hippuric acid—fruits and vegetables; *4*) trimethylamine *N*-oxide (TMAO)—seafood, meat, and eggs; and *5*) tryptophan betaine—nuts/legumes.

**Conclusions:**

Specific serum metabolites reflect intake of citrus fruit/juice, vegetables, animal foods, and nuts/legumes in pregnant women independent of ethnicity, fasting status, and delays to storage across multiple collection centers. Robust biomarkers of overall diet quality varied by cohort. Proline betaine, 3-methylhistidine, hippuric acid, TMAO, and tryptophan betaine were robust dietary biomarkers for investigations of maternal nutrition in diverse populations.

## Teaser

Study identifies robust dietary biomarkers of dietary patterns and foods in pregnant women.

## Introduction

High-throughput metabolomic profiling technology has rapidly advanced clinical medicine in recent years (1). Its application in large-scale epidemiological studies also offers novel insights regarding how dietary exposures influence chronic disease risk (2–4). This approach could shift studies of diet and health away from a reliance on FFQs as the dietary assessment tool of choice for large-scale population-based studies (3, 5). Although FFQs can broadly stratify people as either high or low consumers of certain foods and nutrients (6), they fare less well at estimating exact intakes of many nutrients (7), and can produce biased estimates of true intake because participants rely on memory rather than recording information in real time (8), and their responses are subject to social desirability bias (9). Furthermore, most FFQs lack detailed information on food preparation methods while not reflecting variable rates of digestion and absorption of nutrients via the gastrointestinal tract, and biotransformation by the liver and gut microbiota (5). Food metabolites that are not subject to large interindividual differences in metabolism have great potential to reflect true food consumption more accurately, avoiding the limitations of the self-reported FFQ (5).

Biomarkers of food intake can be sensitive and specific to changes in dietary patterns in free-living populations (10, 11). Previous large studies of food-metabolite associations have been conducted predominantly in white nonpregnant populations, either including only men, or postmenopausal women. It is well described that pregnancy consists of a series of small, continuous physiological changes that affect the metabolism of all nutrients. For example, adjustments in the metabolism of nitrogenous compounds are in place by the second quarter of pregnancy, and these serve to promote positive nitrogen balance during the final quarter of pregnancy when fetal demands are greatest (12). Changes in maternal dietary patterns during gestation can augment the physiological adaptations. However, the substantial variability in food intakes makes it difficult to assess using conventional assessment tools. Though some studies have described metabolic phenotype changes across a healthy pregnancy (13–15) or adverse pregnancy conditions (16, 17), few have reported associations in dietary intake in pregnancy based on circulating metabolites that are generalizable in a multiethnic population.

A recent publication that summarized the results of an NIH-organized workshop on “Omics Approaches to Nutritional Biomarkers” (18) highlights that future work to “test […] a variety of foods and dietary patterns across diverse populations to identify universal candidate biomarkers” is necessary. Thus, replication studies involving candidate dietary biomarkers are key to translational epidemiology that can impact population health (19). Here we report the association between self-reported dietary intake using a semiquantitative FFQ and 47 serum metabolites consistently measured with high frequency in a multiethnic population of 900 pregnant women from 3 independent birth cohorts. Specifically, in pregnant women in their second trimester our objectives were to: *1*) identify serum metabolites associated with maternal adherence to a high- or low-quality diet, and *2*) determine correlations between selected food groups with putative circulating metabolites associated with diet quality across 3 birth cohorts from Canada, appropriately considering variations in ethnicity, regional location, and fasting status.

## Methods

### Participants

This investigation was conducted on data and serum samples obtained from 3 prospective birth cohorts of pregnant women conducted in different geographical regions of Canada, and enrolling women of diverse ethnicities that comprised the NutriGen Birth Cohort Alliance (20). This cohort consortium includes mother-infant pairs from the SouTh Asian biRth cohorT (START) study (21) recruited from the Peel Region (Ontario); the Family Atherosclerosis Monitoring In earLY life (FAMILY) study (22) recruited from the city of Hamilton (Ontario); and the Canadian Healthy Infant Longitudinal Development (CHILD) cohort study (23) recruited from 4 cities/regions across the country (Winnipeg-Morden, Manitoba; Vancouver, British Columbia; Hamilton, Ontario; and Toronto, Ontario). Participants were recruited between 2004 and 2012 and follow-up is ongoing. For more details on these cohorts, please refer to the **Supplemental Methods**.

Detailed dietary information was available from a semiquantitative FFQ from each cohort. The START and FAMILY birth cohorts used FFQs specifically designed and validated for use in Canadian South Asians and white Caucasians, respectively (24). The CHILD study used a “Canadianized” multiethnic FFQ derived from a validated instrument created by the Fred Hutchinson Cancer Research Center (25). We excluded women who did not complete an FFQ sufficiently (>5% of questions left blank), or who reported implausible energy intake (<500 or >6500 kcal/d). This left 5001 eligible for serum metabolomics analyses, of which 900 were selected for the present analyses based on contrasting diet quality, as described below.

### Diet quality assessment

The FFQs were harmonized to create 36 common food groups as described previously (26). Though others have used the Healthy Eating Index (27, 28) or Alternative Healthy Eating Index (AHEI) (29) to characterize dietary patterns, our FFQs did not capture all of the AHEI components with sufficient precision for direct use. We therefore developed a diet quality score (DQS), calculated as the sum of daily servings of “healthy” foods (fermented dairy, fish and seafood, leafy green vegetables, cruciferous vegetables, legumes, fruits, nuts, and whole grains) less the sum of daily servings of “unhealthy foods” (processed meats, refined grains, French fries, snacks, sweets, and sweet drinks), described in **Supplemental Table 1**. These foods were chosen because they have been widely used to characterize healthy dietary patterns (i.e., prudent diet) that reduce chronic disease risk (30–37). Our DQS correlates well with a modified version of the AHEI previously derived in these cohorts (26, 38).

Within each cohort, those with a DQS >90th percentile of the cohort were considered “high” diet quality; those with a DQS <10th percentile of the cohort were considered “low” diet quality, and the remaining participants were considered “intermediate” diet quality. We then selected 100 participants from each of these 3 groups at random from each cohort (300 per cohort), to create a cohort of 900 pregnant women for serum metabolomics analyses (**Supplemental Figure 1**).

The DQS is a single aggregate metric that considers both healthy foods and unhealthy foods, and therefore it could lack specificity for serum metabolites. To address this issue, we separated the diet score into a healthy diet subscore and an unhealthy diet subscore, and reassessed the associations of each individually, and mutually adjusted, with serum metabolites. The subscore is the number of servings for each of the “healthy” and “unhealthy” items in the score. The healthy score is the sum of servings of fermented dairy, fish and seafood, leafy green vegetables, cruciferous vegetables, legumes, fruits, nuts, and whole grains. The unhealthy score is the sum of servings of processed meats, refined grains, French fries, snacks, sweets, and sweet drinks. The DQS ranges from −41.1 to 66.6, after constraining influential leverage points in the components of the DQS (unhealthy and healthy diet scores >3 × IQR were winsorized at the fifth and 95th percentiles). The distribution of these scores is presented in **Supplemental Figures 2–5**.

### Covariates

In addition to ethnicity and whether the sample was collected in the fasting or nonfasting state, and region in Canada (CHILD), we also used maternal age and gestational age at time of recruitment, sociodemographic information, prepregnancy BMI, parity, multivitamin use, smoking history, height, weight, and medical history (including gestational diabetes and hypertension during the current pregnancy) from existing data files.

### Biospecimen collection and metabolomic analysis

Serum samples were collected from all pregnant women and stored in liquid nitrogen at the Hamilton Clinical Research Laboratory. In 2 of the cohorts, the sample was collected after an overnight fast (START, FAMILY) and in 1 a random nonfasting sample was collected (CHILD).

### Maternal serum metabolome analyses

A validated, high-throughput platform based on multisegment injection-capillary electrophoresis-mass spectrometry (MSI-CE-MS) was used for the identification and quantification of polar/ionic metabolites measured consistently in serum filtrate samples with stringent quality control (QC) (10, 39–41). This multiplexed separation platform is described in more detail in the Supplemental Methods, including a standardized method protocol for characterization of the maternal serum metabolome. Briefly, the number of serum metabolites that satisfied selection criteria for analysis in START, FAMILY, and CHILD were 67, 66, and 47, respectively; of these, 47 serum metabolites were measured consistently across all 3 cohorts when using MSI-CE-MS under 2 configurations with positive- and negative-ion mode detection. An iterative data workflow was used to reject spurious signals, redundant peaks, and background ions when performing targeted and nontargeted metabolite profiling based on analysis of a pooled serum sample that also served as QC for assessing technical precision (40). Furthermore, serum metabolites were analyzed only if they satisfied 2 additional criteria: *1*) the metabolite was detectable in ≥75% of individual samples in a cohort (i.e., frequency filter), and *2*) the technical precision for metabolites measured in repeat QC samples (i.e., reproducibility filter) had a CV <30% (or 40% for low-abundance metabolites with signal-to-noise <10). Nondetectable values were replaced with a missing value input corresponding to half of the minimum response measured for a serum metabolite in each cohort. Also, a robust QC-based batch correction algorithm was used to correct for long-term signal drift when using MSI-CE-MS, as described elsewhere (40). In this work, most serum metabolites were unambiguously identified (level 1) by their comigration and accurate mass (<5 ppm) after spiking with an authentic standard in a pooled QC sample, and subsequently quantified (micromolar) using a calibration curve, where ion responses were normalized to a single internal standard (i.e., relative peak area, RPA). Reference concentrations for serum metabolites for second-trimester pregnant women from different birth cohorts are reported elsewhere (). Otherwise, all serum metabolites were annotated based on their characteristic accurate mass and relative migration time (RMT) under positive (p) or negative (n) ion mode (*m/z*:RMT:mode). Also, unknown serum metabolites were further annotated based on their most likely molecular formula (level 4), with most compounds putatively identified (level 2 or 3) following acquisition of high-resolution tandem MS spectra at different collision energies (42). This stringent process ensured that only fully authenticated serum metabolites reliably measured in most serum samples were correlated to habitual dietary patterns to reduce false discoveries and data overfitting.

### Ethics

Enrolled participants provided full informed written consent for participation, and each study obtained ethics approval from the McMaster Hamilton Integrated Research Ethics Board [START (HiREB #10–640), FAMILY (HiREB #02–060), and CHILD (HiREB #07–2929)].

### Statistics

For objective 1 (identification of dietary biomarker candidates), we performed 2-tailed *t* tests to compare mean natural logarithm–transformed metabolite concentrations between pregnant women with low (*n* = 100) compared with high (*n* = 100) diet quality within each cohort. We considered batch-corrected serum metabolite response (RPA) differences nominally significant at *P* < 0.10 (without correction for multiple testing) candidates for multivariate analyses (43). In multivariable linear regression models, natural logarithm serum metabolite RPAs were regressed on the continuous diet score within each cohort (*n*  = 300), adjusted for prepregnancy BMI, gestational age, total energy (kcal), maternal age, maternal ethnicity (in CHILD only, because it was the only cohort with multiple ethnicities), and center (in CHILD only, because it was a multicenter study). DQS-serum metabolite associations were considered significant at *P* < 0.05, tested independently with no correction for multiple testing. To understand whether it was the presence or absence of “healthy” or “unhealthy” foods driving associations, we fit similar multivariate linear regression models for the healthy and unhealthy subscores separately, each additionally adjusted for the opposing diet subscore.

For objective 2 (linking serum metabolites to self-reported habitual intake of specific foods), we selected food groups for which there is moderate to strong evidence of a metabolite biomarker in the published literature, as summarized by Exposome Explorer (http://exposome-explorer.iarc.fr; see Supplemental Methods). Food group variables represented as servings per day were natural log transformed to correct for skewness prior to analysis. Analysis included: *1*) reporting unadjusted pairwise Pearson correlation coefficients between the serum metabolite and specified food group; *2*) assessing the association between serum metabolite concentration and DQS and foods, using multivariable linear regression (using log-transformed metabolites), adjusted for prepregnancy BMI, gestational age, total energy intake, maternal age, and ethnicity (in CHILD, because it was the only cohort with multiple ethnic groups), prior to meta-analysis, and region in Canada (CHILD only—Toronto, Edmonton, Winnipeg, and Vancouver); *3*) combining the results of the 3 cohorts using inverse-variance random-effects meta-analyses; and *4*) for significant diet-metabolite pairs we conducted random-effects metaregressions to explore/evaluate the moderating effect role of sample fasting status.

To assess the robustness of our regression models, we conducted *k*-fold crossvalidation by dividing the dataset into 10 equal-size subsets (i.e., *k* = 10) (44). For each iteration, we combined *k *− 1 subsets to serve as the training set, and the 1 remaining subset served as the test set. Every sample served as a test data point once and only once, with higher Pearson correlation coefficient values (*r*) considered evidence of a robust association. All analyses were completed in R (v3.6.3; R Foundation). Model assumptions and missing data handling are described in the Supplemental Methods.

## Results

### Participant characteristics

Nine hundred participants were included in the discovery analysis (300 women from each of the 3 cohorts) (**Table 1**). Participants in the metabolomics study were generally representative of pregnant women in each of the cohorts with a mean (± SD) age of 31.2 ± 4.7 y, and a prepregnancy BMI of 25.1 ± 5.3 kg/m^2^. All women in START were South Asian; and >97% were white Caucasian in FAMILY and CHILD. White Caucasian women from CHILD were overrepresented in our sample compared with the full CHILD cohort (97.7% compared with 72.9%). Overall, 44% of women were primiparous, >89% used a prenatal vitamin, 79% had never smoked, 18% were former smokers, and 3% were current smokers. All START mothers were lifelong never smokers, and the smoking profiles were similar in CHILD and FAMILY. The cohorts had different chronological and gestational ages at recruitment (with START mothers being youngest, and FAMILY being oldest), and different prepregnancy BMI (with START mothers being lowest, and FAMILY being highest). START mothers were most likely to have gestational diabetes (26.2% of mothers), and least likely to be employed at the time of the survey (54.2%).

**TABLE 1:** Participant characteristics for the metabolomics subcohort analysis^1^

Cohort	START (*n* = 300)	FAMILY (*n* = 300)	CHILD (*n* = 300)	Overall (*n* = 900)
Age, y, mean (SD)	30.0 (3.7)^a^	32.3 (4.9)^b^	31.3 (5.1)^c^	31.2 (4.7)
Gestational age at recruitment, wk, mean (SD)	26.6 (1.7)^a^	29.5 (3.8)^b^	25.1 (6.5)^c^	27.1 (4.8)
Prepregnancy BMI, kg/m^2^, mean (SD)	23.9 (4.3)^ac^	26.8 (6.4)^b^	24.4 (5.0)^c^	25.1 (5.3)
Primiparous, *n* (%)	103 (34.8)	154 (51.3)	134 (45.9)	391 (44.0)^2^
Prenatal multivitamin use, *n* (%)	285 (95.0)	220 (94.0)	290 (96.7)	795 (95.3)^2^
Type 2 diabetes (baseline), *n* (%)	8 (2.7)	9 (3.0)	4 (1.3)	21 (2.3)^2^
Gestational diabetes, *n* (%)	76 (26.2)	50 (17.5)	12 (4.0)	138 (15.8)^2^
Hypertension (baseline), *n* (%)	4 (1.3)	9 (3.0)	9 (3.0)	22 (2.5)^2^
Gestational hypertension, *n* (%)	5 (1.7)	11 (3.7)	6 (2.1)	22 (2.5)^2^
Employed full- or part-time, *n* (%)	162 (54.2)	248 (82.7)	240 (80.5)	650 (72.5)^2^
Maternal ethnicity				
White Caucasian, *n* (%)	0 (0.0)	300 (100.0)	293 (97.7)	593 (65.9)^2^
South Asian, *n* (%)	300 (100.0)	0 (0.0)	1 (0.3)	301 (33.4)^2^
East/Southeast Asian, *n* (%)	0 (0.0)	0 (0.0)	2 (0.7)	2 (0.2)^2^
African, *n* (%)	0 (0.0)	0 (0.0)	1 (0.3)	1 (0.1)^2^
Other, *n* (%)	0 (0.0)	0 (0.0)	3 (1.0)	3 (0.3) ^2^
Smoking history				
Never smoked, *n* (%)	300 (100.0)	191 (64.8)	217 (73.1)	708 (79.4)^2^
Quit before pregnancy, *n* (%)	0 (0.0)	48 (16.3)	56 (18.9)	104 (11.7)^2^
Quit during pregnancy, *n* (%)	0 (0.0)	43 (14.6)	11 (3.7)	54 (6.1)^2^
Current smoker, *n* (%)	0 (0.0)	13 (4.4)	13 (4.4)	26 (2.9)^2^
Diet quality				
Diet quality score	7.1 (8.1)^a^	1.6 (6.5)^b^	3.2 (8.6)^c^	4.0 (8.1)
Healthy foods, servings/d	12.0 (6.2)^a^	9.1 (4.7)^b^	10.1 (6.4)^c^	10.4 (5.9)
Unhealthy foods, servings/d	4.9 (3.0)^a^	7.4 (3.9)^b^	6.9 (4.5)^c^	6.4 (4.0)
mAHEI^3^	36.3 (10.8)^a^	29.6 (10.9)^b^	29.3 (10.3)^c^	31.8 (11.1)

1 Values in same row with different superscript are significantly different (*P*  < 0.05) on Tukey honestly significant difference post hoc test. CHILD, Canadian Healthy Infant Longitudinal Development; FAMILY, Family Atherosclerosis Monitoring In earLY life; mAHEI, Modified Alternative Healthy Eating Index; START, SouTh Asian biRth cohorT study.

2 Distribution of this condition was different across the 3 cohorts.

3 mAHEI scored as follows: *1*) Fruits [4 + servings/d = 10 points; (servings/d divided by 4 × 10) = score]; *2*) Vegetables [5 + servings/d = 10 points; (servings/d divided by 5 × 10) = score]; *3*) Nuts and soy protein [1 + servings/d = 10 points; (servings/d divided by 1 × 10) = score]; *4*) Ratio of fish servings to (meat + eggs servings) >4.0 = 10 points; (ratio/4 × 10) = score); *5*) Whole grains [3 + servings/d = 10 points; (servings/d divided by 3 × 10) = score]; *6*) Fried foods (reverse scored) <0.5 servings = 10 points; (ratio/0.5 × 10) = negative score; 5 + servings/d = −10. Total mAHEI score = sum points (fruits, vegetables, nuts and soy protein, fish:meat + eggs ratio, whole grains, fried foods); maximum = 60, minimum = 0.

The Spearman rank coefficient (ρ) was 0.76 (*P* < 0.0001) between the DQS and the modified Alternative Healthy Eating Index (mAHEI) for the entire data set (*n* = 900), which was consistent across each cohort (*r* = 0.66 in START, 0.76 in FAMILY, and 0.68 in CHILD; *n* = 300 each) (**Table 2**). The mean maternal DQS in pregnancy differed significantly between the cohorts and was highest in START (7.1 ± 8.1), lowest in FAMILY (1.6 ± 6.5) and intermediate in CHILD (3.2 ± 8.6) (Table 1, Supplemental Figures 2–5). Across the cohorts, a higher DQS was consistently associated with higher mAHEI, higher total fiber, protein, vitamin A, vitamin C, folate, calcium, and potassium intakes, a higher polyunsaturated:saturated fat ratio, as well as lower saturated and *trans*-fat and cholesterol intakes (Table 2), reflecting a nutrient-rich and health-promoting maternal dietary pattern.

**TABLE 2:** Dietary intake by diet quality score^1^

	START (*n* = 300)					FAMILY (*n* = 300)					CHILD (*n* = 300)				
Score	Low (*n* = 100)	Medium (*n* = 100)	High (*n* = 100)	*P*-trend^3^	*P*-value H vs. L^4^	Low (*n* = 100)	Medium (*n* = 100)	High (*n* = 100)	*P*-trend	*P*-value H vs. L	Low (*n* = 100)	Medium (*n* = 100)	High (*n* = 100)	*P*-trend	*P*-value H vs. L
Diet quality score	−0.4 (4.9)^2^	6.2 (2.2)	15.6 (6.4)	**<0.0** **001**	**<0.0001**	−5.7 (3.4)	1.7 (2.3)	8.7 (2.5)	**<0.0001**	**<0.0001**	−6.1 (2.4)	2.3 (2.4)	13.4 (4.3)	**<0.0001**	**<0.0001**
Healthy score	6.6 (3.2)	10.6 (3.0)	18.6 (4.5)	**<0.0001**	**<0.0001**	5.6 (2.8)	7.7 (2.9)	13.9 (3.5)	**<0.0001**	**<0.0001**	5.8 (2.9)	6.9 (2.6)	17.5 (5.0)	**<0.0001**	**<0.0001**
Unhealthy score	6.6 (3.2)	4.4 (2.8)	3.5 (2.0)	**<0.0001**	**<0.0001**	11.1 (3.8)	6.0 (2.4)	5.2 (2.2)	**<0.0001**	**<0.0001**	11.9 (4.1)	4.6 (1.8)	4.1 (2.0)	**<0.0001**	**<0.0001**
mAHEI^5^	27.8 (9.2)	36.8 (8.2)	44.4 (7.6)	**<0.0001**	**<0.0001**	20.8 (8.0)	28.0 (8.0)	40.1 (6.2)	**<0.0001**	**<0.0001**	22.3 (8.7)	27.0 (8.0)	38.7 (6.0)	**<0.0001**	**<0.0001**
DQS ρ mAHEI	0.66					0.76					0.68				
Macronutrients															
Energy (kcal)	1812 (737)	1847 (734)	2245 (650)	<0.0001	<0.0001	2482 (816)	1982 (647)	2447 (708)	0.74	**0.74**	2657 (890)	1868 (602)	2279 (703)	**0.024**	0.001
% Carbohydrate	54.4 (5.9)	52.7 (5.5)	54.3 (5.1)	0.99	0.86	55.5 (6.0)	52.4 (5.4)	52.1 (5.9)	**<0.0001**	<0.0001	49.1 (6.6)	48.8 (6.3)	49.7 (6.6)	0.39	**0.79**
Fiber, g	16.6 (7.5)	22.5 (8.4)	34.1 (8.4)	**<0.0001**	**<0.0001**	16.3 (6.6)	17.4 (6.9)	28.3 (8.8)	**<0.0001**	**<0.0001**	24.9 (9.2)	23.7 (8.7)	36.3 (10.2)	**<0.0001**	**<0.0001**
% Protein	14.9 (2.4)	16.7 (2.6)	16.7 (2.5)	**<0.0001**	**<0.0001**	15.0 (2.2)	17.4 (2.7)	18.1 (2.4)	**<0.0001**	**<0.0001**	16.1 (2.8)	17.8 (2.6)	17.4 (3.2)	**<0.02**	**<0.02**
% Fat	30.6 (4.6)	30.5 (4.3)	29.0 (3.9)	**0.002**	**<0.004**	29.0 (4.9)	29.6 (5.0)	29.2 (4.8)	0.73	**0.72**	34.8 (5.4)	33.3 (5.7)	32.9 (6.2)	0.09	0.10
% SFA	10.6 (2.4)	10.8 (2.6)	9.7 (1.8)	**<0.003**	**0.0007**	11.4 (2.3)	11.1 (2.2)	10.2 (2.6)	**0.0009**	<0.002	12.4 (2.3)	11.6 (2.3)	10.7 (2.5)	**<0.0001**	**<0.0001**
% MUFA	11.4 (2.1)	11.1 (1.9)	10.6 (2.1)	**<0.002**	**<0.005**	10.7 (2.1)	10.9 (2.2)	10.8 (2.2)	0.72	0.71	12.5 (2.4)	12.0 (2.5)	12.0 (2.8)	0.39	0.44
% PUFA	5.6 (1.1)	5.7 (1.2)	5.7 (1.3)	0.90	0.70	3.8 (0.8)	4.1 (1.0)	4.3 (0.9)	**<0.0001**	<0.0001	7.0 (1.5)	6.8 (1.5)	7.4 (1.9)	**0.06**	**0.10**
% *trans* fat	0.16 (0.17)	0.11 (0.10)	0.10 (0.09)	**0.0002**	**<0.003**	0.24 (0.19)	0.18 (0.19)	0.14 (0.14)	**<0.0001**	**<0.0001**	1.30 (0.30)	1.13 (0.31)	0.86 (0.26)	**<0.0001**	**<0.0001**
Cholesterol, mg	194 (121)	201 (214)	176 (105)	**<0.003**	**<0.0001**	263 (104)	226 (94)	286 (130)	0.013	<0.02	339 (162)	246 (105)	292 (160)	**0.94**	0.83
P:S ratio	0.56 (0.19)	0.57 (0.22)	0.61 (0.21)	<0.041	<0.02	0.34 (0.08)	0.38 (0.10)	0.45 (0.15)	**<0.0001**	**<0.0001**	0.58 (0.16)	0.60 (0.16)	0.72 (0.24)	**<0.0001**	**<0.0001**
Vitamins															
Vitamin A (RAE)	1545 (792)	2171 (1178)	3770 (1926)	**<0.0001**	**<0.0001**	1368 (580)	1513 (715)	2617 (1621)	**<0.0001**	**<0.0001**	1213 (504)	1085 (526)	1764 (952)	**<0.0001**	**<0.0001**
Folate (DFE), µg	319 (140)	387 (142)	580 (330)	**<0.0001**	**<0.0001**	280 (112)	277 (95)	417 (109)	**<0.0001**	**<0.0001**	685 (263)	569 (205)	720 (256)	<0.0001	**0.0002**
Vitamin C, mg	190 (97)	236 (112)	335 (111)	**<0.0001**	**<0.0001**	215 (163)	174 (85)	251 (91)	**<0.004**	**<0.009**	153 (115)	150 (77)	242 (115)	**<0.0001**	**<0.0001**
Minerals															
Calcium, mg	937 (471)	1094 (431)	1440 (627)	**<0.0001**	**<0.0001**	1456 (685)	1406 (739)	1707 (690)	**<0.0001**	<0.0001	1588 (650)	1242 (581)	1563 (611)	<0.002	**<0.007**
Iron, mg	13 (6)	14 (6)	20 (7)	**<0.0001**	**<0.0001**	16 (6)	14 (6)	20 (6)	**<0.0001**	<0.0001	18 (7)	14 (5)	18 (7)	0.0008	**<0.004**
Potassium, mg	3143 (1276)	3808 (1268)	5345 (1713)	**<0.0001**	**<0.0001**	4059 (1570)	3791 (1334)	5259 (1449)	**<0.0001**	**<0.0001**	3936 (1340)	3337 (1088)	4683 (1411)	**<0.0001**	**<0.0001**
Sodium, mg	2668 (1339)	2923 (1300)	3866 (1765)	**0.0003**	**<0.004**	2888 (1029)	2517 (1247)	3060 (988)	**0.038**	<0.004	4204 (1438)	3057 (1034)	4150 (1502)	<0.0001	<0.0001

1 CHILD, Canadian Healthy Infant Longitudinal Development; DFE, dietary folate equivalents; DQS, diet quality score; FAMILY, Family Atherosclerosis Monitoring In earLY life cohort; H, highest category; HDS, healthy diet score; L, lowest category; mAHEI, modified Alternative Healthy Eating Index (see footnote 4); P:S ratio, polyunsaturated:saturated fat ratio; RAE, retinol activity equivalents; START, SouTh Asian biRth cohorT study; UDS, unhealthy diet score.

2 Values are mean (SD).

3 Regression of DQS [ordinal (0, 1, or 2)] on nutrient (continuous). The total DQS (Pearson *r* = 0.24 in START, *r* = −0.05 in FAMILY, *r* = −0.13 in CHILD) was weakly associated with total energy because it is a difference measure, but each component (HDS and UDS) was associated with total energy. For healthy score, *r* = 0.63 in START, *r* = 0.51 in FAMILY, and *r* = 0.23 in CHILD. For unhealthy score, *r* = 0.61 in START, *r* = 0.64 in FAMILY, and *r* = 0.58 in CHILD. Tests for trend are adjusted for total energy (except values in row marked "energy”).

4 Independent samples *t* test comparing mean value of measure between “high” and “low” diet quality groups; adjusted for total energy (rationale in footnote 2).

5 mAHEI scored as follows: *1*) Fruits [4 + servings/d = 10 points; (servings/d divided by 4 × 10) = score]; *2*) Vegetables [5 + servings/d = 10 points; (servings/d divided by 5 × 10) = score]; *3*) Nuts and soy protein [1 + servings/d = 10 points; (servings/d divided by 1 × 10) = score]; 4) Ratio of fish servings to (meat + eggs servings) >4.0 = 10 points; (ratio/4 × 10) = score; *5*) Whole grains [3 + servings/d = 10 points; (servings/d divided by 3 × 10) = score]; *6*) Fried foods (reverse scored) <0.5 servings = 10 points; (ratio/0.5 × 10) = negative score; 5 + servings/d = −10. Total mAHEI score = sum points (fruits, vegetables, nuts and soy protein, fish:meat + eggs ratio, whole grains, fried foods); maximum = 60, minimum = 0.

### Associations between diet quality index and serum metabolites

Candidate serum metabolites passing the initial *P* < 0.10 threshold using the extreme-ends approach (**Supplemental Tables 2** and **3**) included 14 from START, 14 from FAMILY, and 9 from CHILD. Collectively, these 29 metabolites were then entered into multivariable linear regression models to assess the associations with the DQS, and the healthy and unhealthy indices separately. The initial screen identified significant and high magnitude of differences in serum metabolic responses (RPA) between high and low DQS for methylhistidine, choline, arginine, and tryptophan betaine in START; hippuric acid, hypoxanthine, methylhistidine, and gluconic acid in FAMILY; and hippuric acid, proline betaine, and monomethylarginine in CHILD, as shown in the volcano plots (**Supplemental Figure 6**a–c).

**FIGURE 1:**
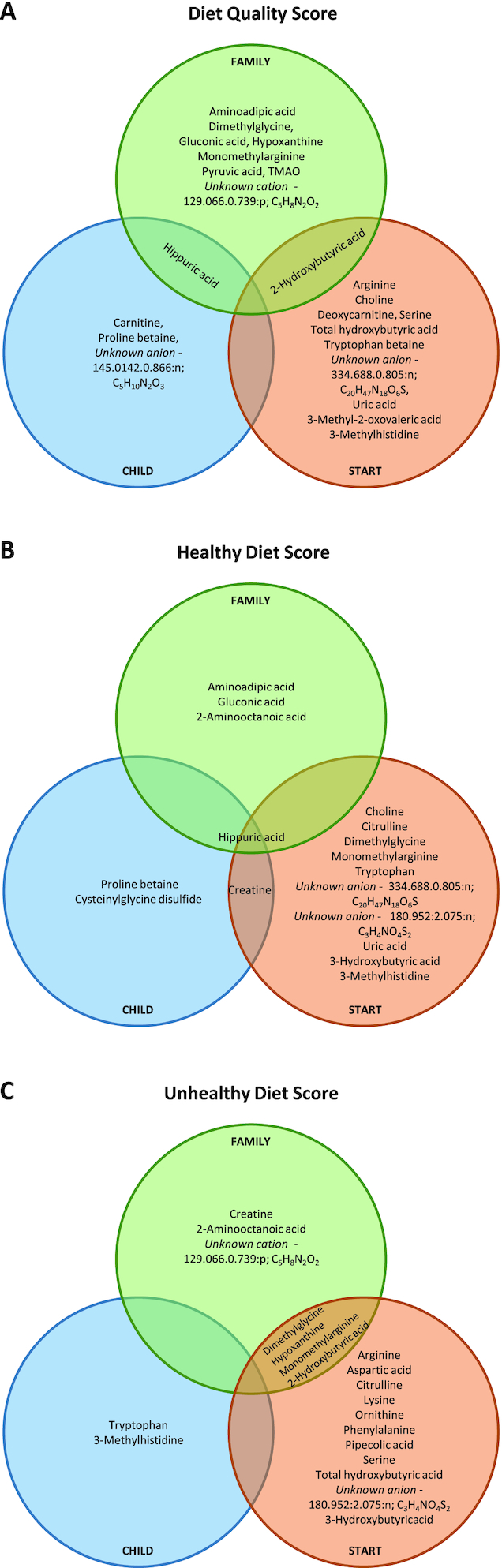
Venn diagram of metabolites that were significantly associated with Diet Quality Score (A); Healthy Diet Score (B); and Unhealthy Diet Score (C); adjusted for adjusted for prepregnancy BMI, gestational age, total energy intake, maternal age, and ethnicity. CHILD, Canadian Healthy Infant Longitudinal Development study; FAMILY, Family Atherosclerosis Monitoring In earLY life study; START, SouTh Asian biRth cohorT study; TMAO, trimethylamine *N*-oxide.

In adjusted multivariate linear models, 8 serum metabolites in START, 10 in FAMILY, and 3 in CHILD were significantly associated with DQS, after adjusting for prepregnancy BMI, maternal age, total energy (kcal), and gestational age (**Supplemental Table 4**). In START, higher DQS was associated with higher circulating concentrations of arginine, choline, serine, tryptophan betaine, 2-hydroxybutyric acid, and an unknown singly charged cation annotated by its *m/z*:RMT:mode and most likely molecular formula [334.688.0.805:p; C_20_H_47_N_18_O_6_S], whereas serum 3-methylhistidine and uric acid were inversely correlated to the DQS. In FAMILY, a higher DQS was associated with higher circulating concentrations of aminoadipic acid, dimethylglycine, gluconic acid, hippuric acid, monomethylarginine, trimethylamine-*N*-oxide (TMAO), and 2-hydroxybutyric acid. In contrast, DQSs were inversely correlated to serum hypoxanthine, pyruvic acid, and a singly charged cation annotated by its *m*/*z*:RMT:mode and most likely molecular formula [129.066.0.739:p; C_5_H_8_N_2_O_2_]. In CHILD, a higher DQS was associated with higher circulating concentrations of hippuric acid, proline betaine, and an unknown singly charged anion [145.0142:0.866:n; C_5_H_10_N_2_O_3_]. However, there was little overlap between the cohorts for these serum metabolites as shown in the Venn diagram, suggesting that though there are some common metabolites, the associations did differ by cohort-level factors (**Figure 1**).

The healthy diet score and unhealthy diet score component analysis within each cohort is shown in Supplemental Table 4. Of the 14 serum metabolites identified in START, 7 (3 positively, 4 negatively) were associated with the healthy diet score and 7 (0 positively, 7 negatively) with the unhealthy diet score. Of the 14 serum metabolites identified in FAMILY, 4 (2 positively, 2 negatively) were associated with a healthy diet score and 6 (2 positively, 4 negatively) with an unhealthy diet score. Of the 9 serum metabolites identified in CHILD, 2 (2 positively, 0 negatively) were associated with healthy diet score and 1 (1 negatively) with an unhealthy diet score. Serum hippuric acid was associated with a healthy diet score in the 2 largely white Caucasian cohorts, FAMILY and CHILD (Supplemental Table 4).

### Associations between specific food groups and serum metabolites

**FIGURE 2:**
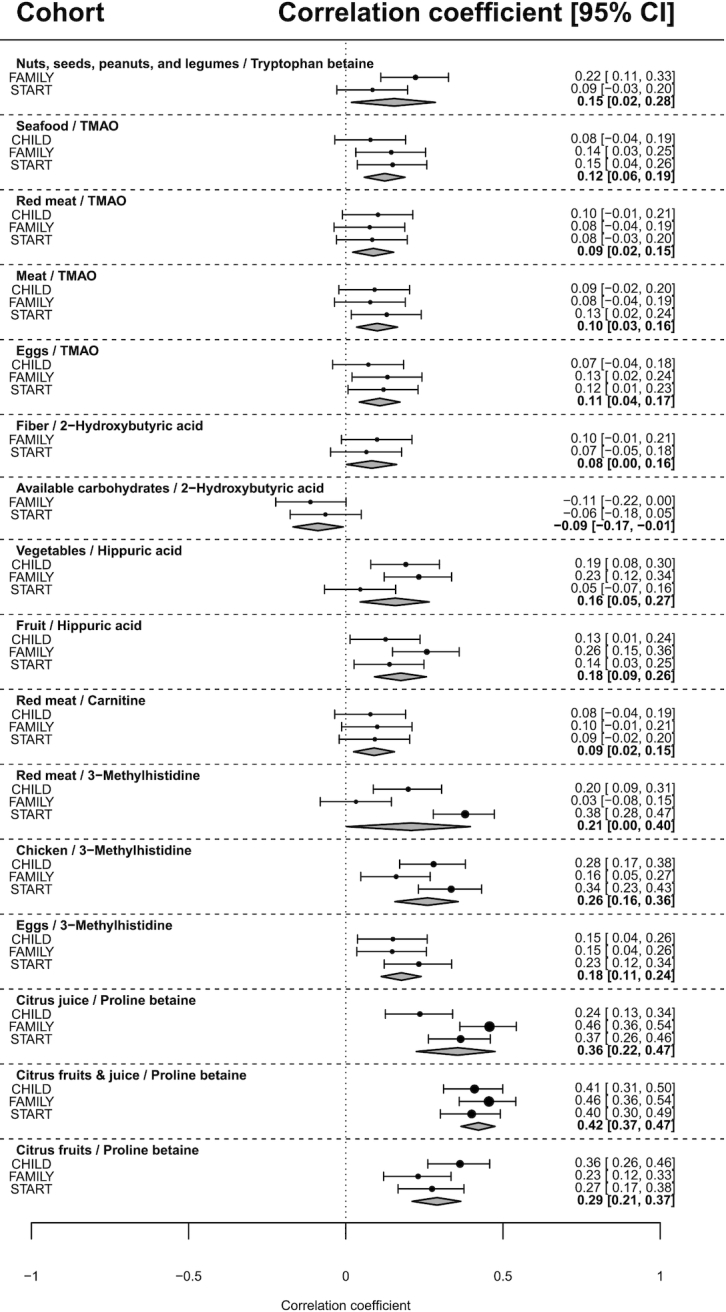
Random effects meta-analyses of metabolite-to-food groups correlations by cohort. Shaded diamonds at the bottom of each meta-analysis shows the pooled point estimate and the left and right vertices are the lower and upport 95% confidence limits. CHILD, Canadian Healthy Infant Longitudinal Development study; FAMILY, Family Atherosclerosis Monitoring In earLY life study; START, SouTh Asian biRth cohorT study; TMAO, trimethylamine *N*-oxide.

**FIGURE 3:**
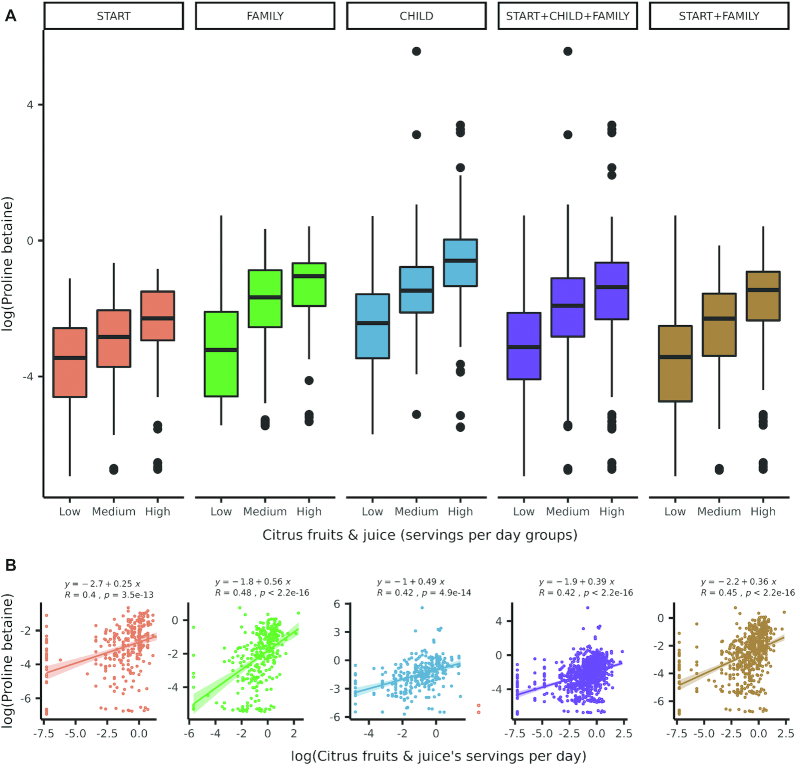
Association of proline betaine with citrus fruits + juice intake by cohort. **Panel A** shows boxplots of the log(Proline Betaine) by cohort, in all cohorts pooled, and in FAMILY and START (the two studies with the most-similarly measured diet) cohorts only, within people with low, medium, and high daily servings of citrus fruits and juice. Whiskers represent 1.5 x interquartile range; black dots denote oulier points. **Panel B** shows linear regression equation (top), and Pearson's R ("R") of proline betaine regressed on log(servings/d of citrus fruits + juices, servings/d) by cohort, in all cohorts pooled, and in FAMILY and START. Solid line is line of best-fit; shading respresents 95 confidence limits of the slope of the regression line. CHILD, Canadian Healthy Infant Longitudinal Development study; FAMILY, Family Atherosclerosis Monitoring In earLY life study; START, SouTh Asian biRth cohorT study.

Robust correlations (i.e., those with a meta-analysis pooled association |>| 0.1 and *P* < 0.05 across all 3 cohorts) were observed between the self-reported intake of several food groups and circulating metabolite concentrations measured in pregnant women (**Figure 2**). Citrus fruits and citrus juice were robustly correlated with serum proline betaine, when grouped together (random effects meta-analysis pooled Pearson *r* = 0.29; *P* < 0.0001; Figures 2 and **3**) and separately for citrus fruits (*r* = 0.42; *P* < 0.0001) and citrus juice (*r* = 0.36; *P* < 0.0001). Red meat (*r *= 0.21; *P* < 0.0001), chicken (*r *= 0.26; *P* < 0.0001), and eggs (*r* = 0.18; *P* < 0.0001) were each positively correlated with serum 3-methylhistidine. Vegetable (*r* = 0.16; *P*  < 0.001) and fruit ( *r* = 0.18; *P* < 0.0001) intake were each positively correlated with serum hippuric acid. Seafood (*r* = 0.12; *P* < 0.0001), meat (*r* = 0.10; *P*  = 0.003), red meat ( *r* = 0.09; *P*  = 0.009), and eggs ( *r* = 0.11; *P*  = 0.001) were each directly associated with serum TMAO concentrations ( **Supplemental Table 5**). Additionally, total intake of nuts, seeds, peanuts, and legumes was modestly correlated with tryptophan betaine (*r* = 0.15; *P *= 0.03). The correlation of the combined intake of nuts, seeds, and peanuts (peanuts were not assessed separately from other nuts and seeds in our cohorts) with tryptophan betaine was nonsignificant, but this metabolite only passed QC for detection in START and FAMILY (*r *= 0.13; *P *= 0.30). Scatterplots of food groups against selected metabolites, and boxplots of the correlation between selected metabolites and food groups by diet quality tertile (i.e., low/medium/high) are presented in **Supplemental Figures 7–11**.

No significant correlations were observed for serum carnitine and total protein or chicken, but a weak association was found with eggs (*r* = 0.06; *P *= 0.06) and red meat (*r* = 0.09; *P *= 0.007). Interestingly, serum uric acid was not associated with consumption of total meat, red meat, chicken, eggs, or total protein. No significant associations were observed for lactate, pyruvate, or 2-hydroxybutyrate and total carbohydrate intake or sugar-sweetened beverages, or for glycine and total protein intake (Supplemental Table 5). Our 10-fold crossvalidation of significantly correlated food item–metabolite pairs yielded poor results (*R^2^*ranging from 0 to 0.16, **Supplemental Table 6**). Model predictions were better in FAMILY than in START and CHILD in most cases, and models pooling our fasting studies (START + FAMILY) performed better than models merging data for fasting and nonfasting studies in 7/8 cases. More complex models combining multiple food items should be tested to improve the prediction with serum metabolites concentrations.

Fasting status influenced the food group to metabolite correlations of uric acid with fruit and vegetables, and lactic acid with carbohydrates. For those serum metabolites, which were significantly correlated with specific foods/food groups, we investigated the influence of fasting status that also coincides with longer delays in blood processing across multiple centers in CHILD by adjusting for this variation in a metaregression (**Supplemental Table 7**).

## Discussion

In this study, we examined self-reported dietary and quantitative metabolomics data from 3 unique cohorts of women in their second trimester of pregnancy. We demonstrate that the serum metabolomic phenotypes can reflect complex dietary patterns when foods are classified as predominantly healthy or unhealthy, and that several serum metabolites are also associated with the average intake of specific food items as applied to a multiethnic Canadian population. The correlations between dietary scores and circulating metabolites are generally modest (*r* ∼0.2–0.4), though robust correlations exist between certain foods and certain serum metabolites that are consistent across cohorts irrespective of fasting status, age, prepregnancy BMI, ethnicity, and/or region.

Maternal metabolism changes substantially during pregnancy. In this diverse cohort of pregnant women from across Canada, we replicated previously described food-metabolite associations, notably citrus-containing fruits and juices with circulating concentrations of proline betaine (Figure 3) because it is an exogenous compound prevalent in citrus juices (45), as well as red meat, chicken, and eggs with methylhistidine because both 1- and 3-methylhistidine positional isomers are present in muscle and other dietary sources of histidine, such as eggs (46). Also, the average intake of vegetables and/or fruit was associated with serum hippuric acid (a major metabolite of flavonoids prevalent in fruits and vegetables) (47), whereas self-reported consumption of seafood, meat, and eggs was also correlated with serum TMAO (present in free form in fish and animal flesh, and also generated from the actions of host and gut microflora cometabolism of carnitine from intake of meat or eggs) (48, 49). Additionally, intake of nuts/legumes was associated with circulating concentrations of tryptophan betaine (which accumulates in the seeds of most *Erythrina* species) (50), and was previously reported to be associated with peanut intake (51). In future investigations of maternal diet and child health outcomes these serum metabolites can be combined to constitute a “metabolic signature” reflecting a healthy diet, and generalizable to a multiethnic population. Specific food group–metabolite associations were robust across cohorts despite differences in DQS distributions in maternal populations sampled from multiple regions across Canada (Figure 2).

Prior investigations of the relation between dietary intake in pregnancy and the serum metabolome are limited. Cross-sectional studies have explored urinary untargeted metabolomics in healthy pregnant women (15), and 2 studies investigated targeted metabolomics longitudinally in healthy pregnancies (14, 52), but neither investigated the relation between a standardized dietary score and blood metabolite profile. In these studies, compared with nonpregnant women, all lipoprotein subclasses and lipids are increased in pregnant women, notably the intermediate-density, low-density, and high-density lipoprotein triglyceride concentrations. Large differences are also seen for many fatty acids and amino acids. Pregnant women also have higher concentrations of low-grade inflammatory marker glycoprotein acetyls and IL-18 and lower concentrations of IL-12p70 (15). The plasma concentrations of several essential and nonessential amino acids, long-chain PUFAs, carnitines, phosphatidylcholines, and sphingomyelins have been reported to change as a function of gestational period (14). Though previous studies have shown that characterization of the human metabolome can reflect differences in contrasting dietary patterns (10, 53), there have been few studies analyzing the maternal serum metabolome during pregnancy (13, 15), and these are limited due to their small size and lack of generalizability to diverse populations without dietary associations to semiquantitative FFQs.

Though there were several consistent food-metabolite associations demonstrated across cohorts, we also highlight some differences. These differences suggest that dietary biomarkers discovered in largely white Caucasian populations of men and postmenopausal women might not transfer to other ethnicities with distinctive dietary patterns, and possibly life-stages, such as major physiological adaptations occurring during pregnancy. These differences can arise for several reasons: *1*) differences in the number of foods within categories on the FFQ (e.g., 20 vegetables on the South Asian FFQ; 18 vegetables on the white Caucasian FFQ); *2*) composition of the foods that make up food groups that contribute to the dietary scores across cohorts (e.g., roti, paratha, and chapatti as sources of whole grains in South Asians compared with whole wheat bread and rolls in white Caucasians); *3*) differences in cooking methods (e.g., potatoes are usually curried with spices or stir-fried among South Asians whereas potatoes are mainly boiled, mashed, or baked among white Caucasians); and/or *4*) between-subject differences in absorption and metabolism of foods and nutrients. Despite efforts to create a standardized DQS that would be associated with presumably similar serum metabolites across cohorts, differences, presumably due to the unique foods eaten by each cohort, led to lack of consistency in the association between the DQS and specific serum metabolites. Another study limitation was that CHILD was the most heterogeneous birth cohort in terms of serum sampling procedures that were performed under nonfasting conditions for pregnant women recruited from multiple centers in Canada. This resulted in fewer and more variable serum metabolites measured within CHILD compared with the 2 other fasting birth cohorts (START, FAMILY), while also introducing confounding from recent dietary intake rather than habitual (i.e., long-term) dietary consumption patterns that better match self-reported FFQs (54).

Few studies have examined metabolomic markers associated with habitual dietary patterns. In a secondary analysis of a controlled feeding study, a classifier using metabolites that differed between diets was able to correctly differentiate between a low-fat (20%), very-low-carbohydrate (10%), and low-glycemic-index diet (glycemic index = 32.9) in 60 of 63 cases (>95% accuracy) (53). A recent analysis of the Dietary Approaches to Stop Hypertension (DASH) trial, comparing the intervention with the control group, showed that the healthy intervention diet levels of proline betaine and tryptophan betaine were significantly higher in the healthy diet group (55). In a randomized controlled trial we conducted (10, 56), fasting plasma and single-spot urinary proline betaine and 3-methylhistidine trajectories differentiated a “Western” from a “Prudent” dietary pattern in nonpregnant, free-living adults following 2 wk of food provision.

Our work provides evidence that metabolomics can be used to assess habitual intake of specific foods applicable to diverse populations of pregnant women with highly variable and complex dietary patterns. By examining associations of food groups with metabolites established in prior studies, we confirm similar associations exist in a multiethnic cohort of pregnant women in their second trimester for citrus fruits and juices, legumes (including peanuts), meat protein, fruit and vegetables, and seafood, eggs, and meat. Proline betaine, tryptophan betaine, TMAO, 3-methylhistidine, and hippuric acid have been identified in previous studies in nonpregnant adult, and adolescent populations. These serum metabolites could be used in future studies as robust dietary biomarkers reflective of healthy and unhealthy diets that complement FFQ assessments. However, dietary biomarkers are not immune to misclassification errors. For example, because our DQS increases (becomes “healthier”) with increased fruits and vegetables, those who eat a lot of fruits and vegetables, but exclude citrus fruits (i.e., enriched with proline betaine), might still be misclassified by biomarker pattern alone. This fact emphasizes that few dietary biomarkers are entirely specific to certain foods and are more often associated with habitual eating patterns of distinctive collections of food categories. Dose–response associations in nutrition are often difficult to demonstrate (57). Here, we show increases in circulating metabolites proportionate to intake of the studied foods within each cohort, and when all 3 cohorts are pooled, as shown in Figure 2 and Supplemental Figures 7–11.

Inferring direct associations between food intake and biomarkers in observational studies is difficult. Indeed, larger studies of metabolomic markers as independent predictors of cardiovascular disease have shown little correlation to the putative (self-reported) food sources of that compound (58). This is likely because the FFQ and the metabolite reflect different time windows of exposure—the FFQ typically the previous year, and the metabolite typically days if not hours, depending on fasting status. Detection of compounds that are largely not produced endogenously, such as proline betaine, indicate high consumption of that food (e.g., citrus) with high certainty. Detection of compounds produced from body protein catabolism and food sources, such as methylhistidine, might not exclusively reflect consumption of that food (e.g., meat) with any certainty. Well-controlled feeding studies are needed to validate the dose–response of putative dietary biomarkers from observational studies (59) in conjunction with characterization of their abundances in various foods to establish their specificity (60). Also, combinations of dietary biomarkers can improve robustness and plausibility instead of single compounds.(61)

### Strengths and limitations

Our work addresses an important limitation in previous metabolomic studies in nutrition by evaluating an understudied population. Maruvada et al. (18) and the NIH group, in addition to noting the importance of testing food intake biomarkers across diverse populations to identify universal candidate biomarkers, also emphasize the importance of “replication of initial biomarker studies in different populations,” which is “often necessary to generalize the results, to accommodate population heterogeneity, and to properly account for food choice diversity and dietary patterns.” This sentiment is echoed by leaders in this field (62), who note “the large inter-individual variation in response to foods mak[es] it difficult to identify biomarkers that respond reproducibly across populations.” Our work involving a multiethnic cohort of pregnant women addresses this major knowledge gap and contributes data on the interpopulation differences in food-metabolite associations.

Although a single independent external study was not selected for replication analysis, the simultaneous assessment of 3 independent birth cohorts from the same country provided independent populations in which to assess consistency, using a validated analytical platform for metabolomic analyses with stringent QC and batch correction adjustment. Limitations include the measurement of diet using a self-reported FFQ; however, this is widely used in epidemiological research studies. Additionally, there was a difference in sample collection timing (i.e., fasting compared with nonfasting) across studies, including delays to processing blood samples across multiple centers. To counter these factors, we used only validated FFQs, and fasting status was adjusted for in the meta-regression analysis. Also, nontargeted metabolite profiling by MSI-CE-MS used in this study was limited to the analysis of polar/ionic metabolites in serum and not lipid classes, which require use of nonaqueous buffer conditions (11). Also, we found that not all metabolites were consistently detectable across cohorts. Finally, we acknowledge that associations in an observational study such as ours are always subject to the possibility of confounding, which is a serious threat to causal inference. We attempted to reduce the likelihood of known confounders through our multivariable adjustment approach, which is also further supported by independent metabolite-dietary associations in nonpregnant populations. However, we cannot exclude the possibility of residual confounding of these associations owing to unmeasured confounders.

### Conclusions

In a multiethnic cohort of pregnant women, DQSs are associated with concentrations of specific circulating serum metabolites that reflect higher intakes of both healthy and unhealthy foods. Proline betaine, 3-methylhistidine, hippuric acid, TMAO, and tryptophan betaine were robust dietary biomarkers associated with habitual intake of specific foods, and can be used for investigations of maternal nutrition in multiethnic populations that can also tolerate preanalytical variations in blood collection in diverse settings (e.g., fasting status, delays to processing).

## Supplementary Material

nzaa144_Supplemental_File

## Notes

The study was funded by the Canadian Institutes of Health Research (CIHR) (Team Grant: DOHaD – Implications for Men, Women, Boys and Girls; 201511). SSA is supported by a Tier 1 Canada Research Chair in Ethnicity and Cardiovascular Disease and Heart and Stroke Foundation Chair in Population Health. PB-M acknowledges support from the Natural Sciences and Engineering Research Council of Canada and Genome Canada. The funders had no role in the study design, data collection and analysis, decision to publish, or preparation of the manuscript.

The datasets generated and/or analyzed during the current study are not publicly available because participants in the FAMILY, CHILD, and START studies did not consent to public sharing of their data at the time of recruitment. Datasets can be made available from the corresponding author on reasonable request.

Author disclosures: RJdS has served as an external resource person to the WHO's Nutrition Guidelines Advisory Group on *trans* fats, saturated fats, and polyunsaturated fats. He has received speaker's fees from the University of Toronto, and McMaster Children's Hospital. He serves as an independent director of the Helderleigh Foundation (Canada). All other authors report no conflicts of interest.

Supplemental Methods, Supplemental Tables 1–7, and Supplemental Figures 1–11 are available from the “Supplementary data” link in the online posting of the article and from the same link in the online table of contents at https://academic.oup.com/cdn/.

RJdS, MS, and AL contributed equally to the preparation of this manuscript.

Abbreviations used: AHEI, Alternative Healthy Eating Index; CHILD, Canadian Healthy Infant Longitudinal Development; DQS, diet quality score; FAMILY, Family Atherosclerosis Monitoring In earLY life; HiREB, Hamilton Integrated Research Ethics Board; mAHEI, Modified Alternative Healthy Eating Index; MSI-CE-MS, multisegment injection-capillary electrophoresis-mass spectrometry; QC, quality control; RMT, relative migration time; RPA, relative peak area; START, SouTh Asian biRth cohorT; TMAO, trimethylamine *N*-oxide.
