# Supplementary material for: Maternal Diet and the Serum Metabolome in Pregnancy: Robust Dietary Biomarkers Generalizable to a Multiethnic Birth Cohort
Source: Curr Dev Nutr. 2020 Sep 2;4(10):nzaa144. doi: 10.1093/cdn/nzaa144 (PMC7547851; doi:10.1093/cdn/nzaa144)
Supplement: nzaa144_Supplemental_File [file nzaa144_supplemental_file.docx]

Contents

[Detailed Methods 4](#_Toc47530021)

[Cohort details 4](#_Toc47530022)

[Serum metabolomics by multisegment injection-capillary electrophoresis-mass spectrometry 5](#_Toc47530023)

[Quality control 5](#_Toc47530024)

[Detailed statistical methods 6](#_Toc47530025)

[Supplementary Tables 8](#_Toc47530026)

[Supplementary Table 1: Algorithm for assigning “healthy” and “unhealthy” diet scores 8](#_Toc47530027)

[Supplementary Table 2a-c: Differences in mean serum metabolite responses between high and low diet quality groups in each of the three birth cohorts. 9](#_Toc47530028)

[Supplementary Table 2a: START cohort (n=200; 100 high vs. 100 low diet quality). 9](#_Toc47530029)

[Supplementary Table 2b: FAMILY cohort (n=200; 100 high vs. 100 low diet quality). 10](#_Toc47530030)

[Supplementary Table 2c: CHILD cohort (n=200; 100 high vs. 100 low diet quality). 11](#_Toc47530031)

[Supplementary Table 3: Summary of multivariate linear regression of cohort-specific metabolites on diet score (DQS). 12](#_Toc47530032)

[Supplementary Table 4: Cohort-specific candidate metabolites significant on overall diet score (DQS), healthy diet subscore (HDS), and unhealthy diet subscore (UDS). 13](#_Toc47530033)

[Supplementary Table 5. Correlations and 95% CI of Specified Metabolite-to-Food Group pairs by cohort with meta-analysis results. 15](#_Toc47530034)

[Supplementary Table 6. Results of *k*-fold cross-validation for nutrient-metabolite associations (*k*=10). 18](#_Toc47530035)

[Supplementary Table 7. Meta-regression summary for serum metabolite-food pairs that exhibited heterogeneity of effects upon meta-analysis 19](#_Toc47530036)

[Supplementary Figures 20](#_Toc47530037)

[Supplementary Figure 1. Flowchart of study participant selection for metabolomic analyses from three ongoing birth cohorts 20](#_Toc47530038)

[Supplementary Figure 2. Histograms of diet quality score. 21](#_Toc47530039)

[Supplementary Figure 3. Histograms of healthy diet score. 22](#_Toc47530040)

[Supplementary Figure 4. Histograms of unhealthy diet score. 23](#_Toc47530041)

[Supplementary Figure 5. Box plots of healthy diet sub score (A), unhealthy diet sub score (B), and diet quality score (C) by cohort. 24](#_Toc47530042)

[Supplementary Figure 6 a-c. Volcano plot of results of t-test screen of mean serum metabolite responses versus high & low diet quality for three maternal birth cohorts. 25](#_Toc47530043)

[Supplementary Figure 6a. START 25](#_Toc47530044)

[Supplementary Figure 6b. FAMILY 25](#_Toc47530045)

[Supplementary Figure 6c. CHILD 26](#_Toc47530046)

[Supplementary Figure 7. Association of TMAO with fish and seafood intake by cohort. 27](#_Toc47530047)

[Supplementary Figure 8. Association of carnitine with red meat intake by cohort 28](#_Toc47530048)

[Supplementary Figure 9. Association of hippuric acid with fruit intake by cohort. 29](#_Toc47530049)

[Supplementary Figure 10. Association of tryptophan betaine with nuts and legume intake by cohort. 30](#_Toc47530050)

[Supplementary Figure 11. Association of 3-methylhistidine with egg intake by cohort. 31](#_Toc47530051)

[REFERENCES 32](#_Toc47530052)

# Detailed Methods

## Cohort details

The Family Atherosclerosis Monitoring in Early Life (FAMILY) cohort is a prospective birth cohort which includes predominantly white Caucasian mothers and their offspring and was designed to understand the early life determinants of risk factors for CVD. FAMILY includes 857 mothers and 901 infants recruited from Southwestern Ontario between 2004 and 2009. Of the mother–infant dyads enrolled during pregnancy,>97 % pairs have provided infant measures at age 1 year and >95 % have provided child measures at age 3 years(1, 2).

The CHILD study is a four center (Vancouver, Edmonton, Winnipeg and Toronto, Canada) longitudinal, population-based birth-cohort study which enrolled 3455 mother–child pairs between 2008 and 2012 with planned 5-year follow-up(3). The focus of CHILD is to identify environmental and genetic determinants of allergic disorders and asthma. The follow-up to age 5 years is complete with >95 % retention to age 1 year, >93 % to age 3 year and >93 % to age 5 years(3, 4).

The South Asian Birth Cohort (START) study aimed to enroll 1000 South Asian mother–child pairs from the greater Toronto area, in the province of Ontario(5).Two sister cohorts recruiting 500 mother–child dyads are underway in rural and urban Bangalore, India. START will study the influence of diverse environments, genetics and epigenetic marks on early life adiposity, growth trajectory and cardio-metabolic factors. Recruitment in Canada began in July 2011, and presently 1012 mothers with 1002 newborns delivered have been enrolled. Follow-up is >95 % complete to age 1and >92 % to age 3 with follow-up visits ongoing.

## Serum metabolomics by multisegment injection-capillary electrophoresis-mass spectrometry

Multisegment injection-capillary electrophoresis-mass spectrometry (MSI-CE-MS) has been previously validated as a multiplexed separation platform that improves sample throughput with data fidelity that is optimal for analysis of polar/ionic metabolites from volume-restricted biospecimens (6-8). In this case, mass spectral information is encoded temporally within a separation as required for unambiguous metabolite authentication and biomarker identification in nontargeted metabolomics (6). In this work, MSI-CE-MS used a seven sample segment serial injection format was used in MSI-CE-MS, where every run comprised a randomized injection of five serum filtrate samples from pregnant women, as well as a pooled serum as QC, and blank extract to assess long-term technical precision and confirm lack of sample carry-over(9). Where maternal samples had non-detectable quantities for a metabolite (< 5% of total samples analyzed), a missing input was used using ½ the lowest ion response ratio. Each sample was analyzed under acidic buffer (pH 1.8) conditions with positive ion mode, and alkaline buffer (pH 8.5) conditions with negative ion mode when using MSI-CE-MS with full-scan data acquisition using a time-of-flight-mass spectrometer, which was optimal for resolution and detection of cationic and anionic serum metabolites, respectively. Further experimental details on MSI-CE-MS and a detailed description of the targeted and nontargeted data workflow applied to characterization of the maternal serum metabolome from pregnant women in this study is described elsewhere(9).

## Quality control

QC samples allow for monitoring instrumental drift and technical variation, which also allows for robust batch-correction that is essential in large-scale MS-based metabolomic studies. QC samples were prepared by pooling serum samples from the START cohort (n = 300), which was used throughout the entire study. In this case, every run in MSI-CE-included a blank filtrate sample to monitor for potential sample carry-over, and a QC introduced in a randomized position for effective between batch-correction when analyzing each cohort of maternal serum samples intermittently over a 7 month period(9). Subsequently, serum metabolites were selected for further statistical analysis if they were detected with high frequency (> 75%) in each cohort together with adequate technical precision (median CV of QCs < 30-40%).

## Detailed statistical methods

We verified regression assumptions and influential leverage points in the diet score were constrained (>3 x IQR were winsorized at the 5^th^ and 95^th^ percentiles). After this the total range of the diet score in the three cohorts ranged from -21.5 (i.e. over 21 more unhealthy food servings/d than healthy food servings/d) to 34.6 (i.e. over 34 more healthy food servings/d than unhealthy food servings). In multivariate models, cohort-specific mean values replaced missing values of pre-pregnancy BMI (n=5 in START; n=3 in FAMILY; n= 36 in CHILD), and gestational age (n=9 in CHILD).

We considered those biomarkers studied through a controlled feeding study, that measured the metabolite response to feeding, in the same tissue (e.g. serum), in at least 2 different studies as “strong” evidence; and those biomarkers studied in a controlled feeding study, and replicated in an observational study as “moderate” evidence. Thirteen food metabolites identified were available in our cohort-specific candidate metabolites.

All analyses were completed in R (v3.4.2) (package *car* (v3.0) was used for evaluating linear regression assumption diagnostics and package *metafor* (v2.0) was used for conducting meta-analyses of Pearson correlation coefficients & the subsequent meta-regressions.

# Supplementary Tables

Supplementary Table 1: Algorithm for assigning “healthy” and “unhealthy” diet scores. Each daily serving of food listed in the category either added a point to the “healthy” score or the “unhealthy” score. The Diet Quality Score (DQS) was the difference between the total “healthy diet points” and the “unhealthy diet points”.

| **Healthy diet component** | **Healthy diet points** | **Unhealthy diet component** | **Unhealthy diet points** |
| --- | --- | --- | --- |
| fermented dairy  fish and seafood  leafy green vegetables cruciferous vegetables  legumes  fruits  nuts  whole grains | +1 per serving (1 c)  +1 per serving (3 oz)  +1 per serving (1/2 c)  +1 per serving (1/2 c)  +1 per serving (1/2 c)  +1 per serving (1 piece)  +1 per serving (1 oz)  +1 per serving (30 g) | processed meats  refined grains  French fries  snacks  sweets  sweet drinks | +1 per serving (3 oz)  +1 per serving (30 g)  +1 per serving (1/2 c)  +1 per serving (1 item)  +1 per serving (1 item)  +1 per serving (1 c) |

Supplementary Table 2a-c: Differences in mean serum metabolite responses between high and low diet quality groups in each of the three birth cohorts.

### Supplementary Table 2a: START cohort (n=200; 100 high vs. 100 low diet quality).

|  | **Metabolite^1^** | ***t-***  **statistic** | **P-value** | **Difference in means (95%CI)** | **Fold-change** |
| --- | --- | --- | --- | --- | --- |
| 1 | Tryptophan betaine | 3.15 | 0.002 | 0.71 | 0.81 |
| 2 | Choline | 3.12 | 0.002 | 0.23 | 1.47 |
| 3 | Arginine | 2.86 | 0.005 | 0.13 | 1.16 |
| 4 | *Unknown cation* 334.688.0.805:p; C_20_H_47_N_18_O_6_S | 2.78 | 0.006 | 0.72 | 0.87 |
| 5 | 3-Methylhistidine | -2.36 | 0.019 | -0.24 | 1.08 |
| 6 | Serine | 2.31 | 0.022 | 0.08 | -3.73 |
| 7 | 2-Hydroxybutyric acid | 2.29 | 0.023 | 0.11 | 0.88 |
| 8 | Phenylalanine | 2.23 | 0.027 | 0.07 | 1.12 |
| 9 | Aminooctanoic acid | 2.11 | 0.036 | 0.12 | 0.97 |
| 10 | Oxoproline | 2.10 | 0.037 | 0.10 | 0.83 |
| 11 | Hypoxanthine | 1.95 | 0.052 | 0.11 | 0.94 |
| 12 | Histidine | 1.93 | 0.054 | 0.06 | 1.19 |
| 13 | Asparagine | 1.68 | 0.095 | 0.06 | 0.92 |
| 14 | Asymmetric dimethylarginine | 1.66 | 0.099 | 0.13 | 0.97 |

**^1^** Only metabolites meeting nominal statistical significance threshold of p<0.10 shown. Unknown serum metabolites annotated by their accurate mass, relative migration time and ionization mode (*m/z*:RMT:mode) and most likely molecular formula; t-statistic testing the null hypothesis that concentrations are equal between high (>90^th^ percentile) (high diet quality) and low diet quality (<10^th^ percentile) groups; difference in means = difference in concentrations (high minus low); fold-change: relative concentration difference between groups.

### Supplementary Table 2b: FAMILY cohort (n=200; 100 high vs. 100 low diet quality).

|  | **Metabolite^1^** | ***t-***  **statistic** | **P-value** | **Difference in means (95%CI)** | **Fold-change** |
| --- | --- | --- | --- | --- | --- |
| 1 | Hippuric acid | 5.18 | 0.000 | 0.50 | 0.79 |
| 2 | Dimethylglycine | 3.35 | 0.001 | 0.14 | 0.92 |
| 3 | Hypoxanthine | -3.15 | 0.002 | -0.15 | 1.09 |
| 4 | Hydroxybutyric acid | 3.01 | 0.003 | 0.16 | 0.82 |
| 5 | Aminoadipic acid | 3.01 | 0.003 | 0.14 | 0.97 |
| 6 | TMAO | 3.01 | 0.003 | 0.29 | 0.89 |
| 7 | Monomethylarginine | 2.78 | 0.006 | 0.17 | 0.93 |
| 8 | 3-Methylhistidine | 2.42 | 0.017 | 0.20 | 0.91 |
| 9 | Proline betaine | 2.39 | 0.018 | 0.52 | 0.79 |
| 10 | Gluconic acid | -2.32 | 0.021 | -0.09 | 1.05 |
| 11 | Lactic acid | -2.11 | 0.036 | -0.08 | 0.98 |
| 12 | *Unknown cation* 129.066.0.739:p; C_5_H_8_N_2_O_2_ | -2.03 | 0.044 | -0.11 | 1.04 |
| 13 | Glutamic acid | -1.94 | 0.053 | -0.08 | 0.80 |
| 14 | Pyruvic acid | -1.78 | 0.077 | -0.09 | 0.61 |

**^1^** Only metabolites meeting nominal statistical significance threshold of p<0.10 shown. Unknown serum metabolites annotated by their accurate mass, relative migration time and ionization mode (*m/z*:RMT:mode) and most likely molecular formula; t-statistic testing the null hypothesis that concentrations are equal between high (>90^th^ percentile) (high diet quality) and low diet quality (<10^th^ percentile) groups; difference in means = difference in concentrations (high minus low); fold-change: relative concentration difference between groups.

### Supplementary Table 2c: CHILD cohort (n=200; 100 high vs. 100 low diet quality).

|  | **Metabolite^1^** | ***t-***  **statistic** | **P-value** | **Difference in means (95%CI)** | **Fold-change** |
| --- | --- | --- | --- | --- | --- |
| 1 | Hippuric acid | 4.50 | 0.000 | 0.67 | 0.75 |
| 2 | Proline betaine | 3.29 | 0.001 | 0.69 | 0.63 |
| 3 | *Unknown anion*  145.0142.0.866:n; C_5_H_10_N_2_O_3_ | 3.13 | 0.002 | 0.09 | 1.06 |
| 4 | 3-Methylhistidine | 2.04 | 0.043 | 0.20 | 0.92 |
| 5 | Uric acid | 1.99 | 0.048 | 0.20 | 1.17 |
| 6 | Monomethylarginine | -1.86 | 0.064 | -0.12 | 1.05 |
| 7 | Glucose | 1.80 | 0.074 | 0.19 | 0.84 |
| 8 | 2-Aminooctanoic acid | 1.72 | 0.088 | 0.11 | 0.97 |
| 9 | Guanidinoacetic acid | 1.67 | 0.097 | 0.23 | 0.95 |

**^1^** Only metabolites meeting nominal statistical significance threshold of p<0.10 shown. Unknown serum metabolites annotated by their accurate mass, relative migration time and ionization mode (*m/z*:RMT:mode) and most likely molecular formula; t-statistic testing the null hypothesis that concentrations are equal between high (>90^th^ percentile) (high diet quality) and low diet quality (<10^th^ percentile) groups; difference in means = difference in concentrations (high minus low); fold-change: relative concentration difference between groups.

Supplementary Table 3: Summary of multivariate linear regression of cohort-specific metabolites on diet score (DQS).

| No. | Metabolite name | START | FAMILY | CHILD |
| --- | --- | --- | --- | --- |
| 1 | Aminoadipic acid |  | ✓+ | n/a |
| 2 | Arginine | ✓+ |  |  |
| 3 | Asparagine |  |  |  |
| 4 | Asymmetric dimethyl arginine |  |  | n/a |
| 5 | Choline | ✓+ |  |  |
| 6 | Dimethylglycine |  | ✓+ |  |
| 7 | Gluconic acid |  | ✓- | n/a |
| 8 | Glucose |  |  |  |
| 9 | Glutamic acid |  |  |  |
| 10 | Guanidineacetic acid |  |  |  |
| 11 | Hippuric acid |  | ✓+ | ✓+ |
| 12 | Histidine |  |  |  |
| 13 | Hypoxanthine |  | ✓- |  |
| 14 | Lactic acid |  |  |  |
| 15 | Monomethylarginine |  | ✓+ |  |
| 16 | Oxo-proline |  |  |  |
| 17 | Phenylalanine |  |  |  |
| 18 | Proline betaine |  |  | ✓+ |
| 19 | Pyruvic acid |  | ✓- | n/a |
| 20 | Serine | ✓+ |  |  |
| 21 | TMAO |  | ✓+ |  |
| 22 | Tryptophan betaine | ✓+ |  | n/a |
| 23 | *Unknown cation* 129.066.0.739:p; C_5_H_8_N_2_O_2_ |  | ✓- |  |
| 24 | *Unknown cation* 334.688.0.805:p; C_20_H_47_N_18_O_6_S | ✓+ | n/a | n/a |
| 25 | *Unknown anion*  145.0142.0.866:n; C_5_H_10_N_2_O_3_ |  |  | ✓+ |
| 26 | Uric acid | ✓- |  |  |
| 27 | 2-Aminooctanoic acid |  |  |  |
| 28 | 2-Hydroxybutyric acid | ✓+ | ✓+ | n/a |
| 29 | 3-Methylhistidine | ✓- |  |  |

✓+ = positively associated with the score at P<0.05, ✓- =negatively associated with the score at P<0.05, n/a indicates the metabolite was not detected in the cohort.

Supplementary Table 4: Cohort-specific candidate metabolites significant on overall diet score (DQS), healthy diet subscore (HDS), and unhealthy diet subscore (UDS).

|  | **DQS** | | | **HDS** | | | **UDS** | | |
| --- | --- | --- | --- | --- | --- | --- | --- | --- | --- |
| **Metabolite names** | **START** | **FAMILY** | **CHILD** | **START** | **FAMILY** | **CHILD** | **START** | **FAMILY** | **CHILD** |
| Aminoadipic acid |  | ✓+ | n/a |  | ✓+ | n/a |  |  | n/a |
| Arginine | ✓+ |  |  |  |  |  | ✓- |  |  |
| Asparagine |  |  |  |  |  |  |  |  |  |
| Asymmetric dimethyl arginine |  |  | n/a |  |  | n/a |  |  | n/a |
| Choline | ✓+ |  |  | ✓+ |  |  |  |  |  |
| Dimethylglycine |  | ✓+ |  | ✓- |  |  | ✓- | ✓- |  |
| Gluconic acid |  | ✓+ | n/a |  | ✓- | n/a |  |  | n/a |
| Glucose |  |  |  |  |  |  |  |  |  |
| Glutamic acid |  |  |  |  |  |  |  |  |  |
| Guanidineacetic acid |  |  |  |  |  |  |  |  |  |
| Hippuric acid |  | ✓+ | ✓+ | ✓+ | ✓+ | ✓+ |  |  |  |
| Histidine |  |  |  |  |  |  |  |  |  |
| Hypoxanthine |  | ✓- |  |  |  |  | ✓- | ✓+ |  |
| Lactic acid |  |  |  |  |  |  |  |  |  |
| Monomethylarginine |  | ✓+ |  | ✓- |  |  | ✓- | ✓- |  |
| Oxo-proline |  |  |  |  |  |  |  |  |  |
| Phenylalanine |  |  |  |  |  |  | ✓- |  |  |
| Proline betaine |  |  | ✓+ |  |  | ✓+ |  |  |  |
| Pyruvic acid |  | ✓- | n/a |  |  | n/a |  |  | n/a |
| Serine | ✓+ |  |  |  |  |  | ✓- |  |  |
| TMAO |  | ✓+ |  |  |  |  |  |  |  |
| Tryptophan betaine | ✓+ |  | n/a |  |  | n/a |  |  | n/a |
| *Unknown cation* 129.066.0.739:p; C_5_H_8_N_2_O_2_ |  | ✓- |  |  |  |  |  | ✓+ |  |
| *Unknown anion* 334.688.0.805:n; C_20_H_47_N_18_O_6_S | ✓+ | n/a | n/a | ✓+ | n/a | n/a |  | n/a | n/a |
| *Unknown anion*  145.0142.0.866:n; C_5_H_10_N_2_O_3_ |  |  | ✓+ |  |  |  |  |  |  |
| Uric acid | ✓- |  |  | ✓- |  |  |  |  |  |
| 2-Aminooctanoic acid |  |  |  |  | ✓- |  |  |  |  |
| 2-Hydroxybutyric acid | ✓+ |  | n/a |  |  | n/a | ✓- | ✓- | n/a |
| 3-Methylhistidine | ✓- | ✓- |  | ✓- |  |  |  | ✓- | ✓- |
| ✓+/✓- | 6/2 | 6/4 | 3/0 | 3/4 | 2/2 | 2/0 | 0/7 | 2/4 | 0/1 |

✓+ = positively associated with the score at P<0.05, ✓- =negatively associated with the score at P<0.05. Checkmarks (✓or ✓) = P<0.05 indicate significant associations in multivariate linear regression of cohort-specific metabolites on diet score (DS), healthy diet sub score (HDS), and unhealthy diet sub score (UDS), adjusted for pre-pregnancy BMI and gestational age (and maternal ethnicity and center in CHILD). For HDS and UDS MLRs, diet score-metabolite associations adjusted for other diet sub scores. n/a indicates that the metabolite was not available for the specific study. n/a indicates the metabolite was not detected in the cohort.

Supplementary Table 5. Correlations and 95% CI of Specified Metabolite-to-Food Group pairs by cohort with meta-analysis results.

| **Specified food intake biomarker (FIB)** | **Food group / item** | **START** | **FAMILY** | **CHILD** | **Random-effects**  **Meta-analysis^1^** |
| --- | --- | --- | --- | --- | --- |
|  |  | **r [95% CI]**  **p-value** | **r [95% CI]**  **p-value** | **r [95% CI]**  **p-value** |  |
| 1)  Proline betaine | Citrus fruit & juice (servings/d) | 0.27 [0.17,0.38]  <0.0001 | 0.23 [0.12,0.33]  <0.0001 | 0.36 [0.26,0.46]  <0.0001 | 0.29 [0.21,0.37] |
|  | Citrus fruit (servings /d) | 0.40 [0.30,0.49]  <0.0001 | 0.46 [0.36,0.54]  <0.0001 | 0.41 [0.31,0.50]  <0.0001 | 0.42 [0.37,0.47] |
|  | Citrus juice (servings /d) | 0.37 [0.26,0.46]  <0.0001 | 0.46 [0.36,0.54]  <0.0001 | 0.24 [0.13,0.34]  <0.0001 | 0.36 [0.22,0.47]* |
| 2) 3-Methylhistidine | Red meat(servings/d) | 0.38 [0.28, 0.47]  <0.0001 | 0.03 [-0.08,0.15]  0.70 | 0.20 [0.09,0.32]  <0.005 | 0.21 [0.00,0.42] |
|  | Chicken(servings/d) | 0.35 [0.23,0.43]  <0.0001 | 0.16 [0.05,0.27]  <0.007 | 0.28 [0.17,0.38]  <0.0001 | 0.26 [0.16,0.36]* |
|  | Eggs(servings/d) | 0.23 [0.12,0.34]  <0.0001 | 0.15 [0.04,0.26]  0.01 | 0.15 [0.04,0.26]  0.004 | 0.18 [0.11,0.24] |
|  | Total protein intake(g/d) | -0.03 [-0.14,0.09]  <0.0001 | 0.14 [0.03,0.25]  0.13 | 0.21 [0.10,0.32]  0.02 | 0.11 [-0.03,0.25]* |
| 3) Carnitine | Red meat(servings/d) | 0.09 [-0.02,0.20]  0.12 | 0.10 [-0.01,0.21]  0.11 | 0.08 [-0.04,0.19]  0.15 | 0.09 [0.02,0.15] |
|  | Eggs(servings/d) | 0.00 [-0.12,0.11]  0.62 | 0.10 [-0.01,0.21]  0.10 | 0.09 [-0.02,0.21]  0.17 | 0.06 [0.00,0.13] |
|  | Total protein intake(g/d) | -0.02 [-0.13,0.09]  0.19 | 0.08 [-0.03,0.19]  0.84 | 0.10 [-0.02,0.21]  0.54 | 0.05 [-0.02, 0.12] |
| 5) Uric acid | Meat(servings/d) | 0.04 [-0.07,0.15]  0.42 | 0.07 [-0.05,0.18]  0.65 | 0.00 [-0.11,0.12]  0.90 | 0.04 [-0.03, 0.10] |
|  | Eggs(servings/d) | 0.04 [-0.08,0.15]  0.52 | 0.05 [-0.06,0.16]  0.53 | 0.01 [-0.11,0.12]  0.92 | 0.03 [-0.03, 0.10] |
|  | Dairy(servings/d) | -0.13 [-0.24, -0.02]  0.04 | -0.01 [-0.13,0.10]  0.60 | 0.03 [-0.09,0.14]  0.63 | -0.04 [-0.13, 0.05]* |
|  | Fruit(servings/d) | -0.10 [-0.21,0.02]  0.09 | -0.02 [-0.13,0.09]  0.28 | 0.09 [-0.02,0.21]  0.12 | -0.01 [-0.12,0.10]* |
|  | Vegetables(servings/d) | -0.09 [-0.20,0.02]  0.11 | -0.05 [-0.16,0.07]  0.39 | 0.06 [-0.05,0.17]  0.24 | -0.02 [-0.11,0.06] |
|  | Fibre (g/d) | -0.09 [-0.20,0.03]  0.28 | -0.06 [-0.17,0.06]  0.20 | 0.05 [-0.06,0.16]  0.53 | -0.03 [-0.11,0.05] |
|  | Total carbohydrates intake(g/d) | -0.03 [-0.14,0.08]  0.86 | 0.04 [-0.07,0.16]  0.42 | -0.05 [-0.16,0.06]  0.64 | -0.01 [-0.08,0.05] |
|  | Available carbohydrates(g/d) | -0.01 [-0.13,0.10]  0.93 | 0.05 [-0.06,0.17]  0.46 | -0.07 [-0.18,0.05]  0.57 | -0.01 [-0.08,0.06] |
| 6) Hippuric acid | Tea(servings/d) | 0.05 [-0.06,0.16]  0.39 | -0.01 [-0.12,0.11]  0.77 | 0.03 [-0.08,0.15]  0.26 | 0.03 [-0.04,0.09] |
|  | Vegetables(servings/d) | 0.05 [-0.07,0.16]  0.53 | 0.23 [0.12,0.34]  <0.0001 | 0.19 [0.08,0.30]  <0.0001 | 0.16 [0.05,0.27]* |
|  | Fruit(servings/d) | 0.14 [0.03,0.25]  0.03 | 0.26 [0.15,0.36]  <0.0001 | 0.13 [0.01,0.24]  0.005 | 0.18 [0.09,0.26] |
| 7) Lactate | Total carbohydrates intake(g/d) | 0.13 [0.02,0.24]  0.17 | 0.00 [-0.12,0.11]  0.35 | -0.14 [-0.25, -0.03]  0.36 | 0.00 [-0.16,0.15]* |
|  | Available carbohydrates(g/d) | 0.11 [-0.01,0.22]  0.20 | 0.03 [-0.08,0.14]  0.26 | -0.14 [-0.25, -0.03]  0.38 | 0.00 [-0.14,0.14]* |
|  | Fibre (g/d) | 0.13 [0.01, 0.24]  0.04 | -0.18 [-0.28, -0.06]  0.08 | -0.04 [-0.15, 0.07]  0.44 | -0.03 [-0.20, 0.14]* |
|  | Sugar sweetened beverages and fruit juice(servings/d) | -0.05 [-0.17,0.06]  0.46 | 0.08 [-0.03,0.19]  0.09 | -0.14 [-0.25, -0.03]  0.04 | -0.04 [-0.17,0.09]* |
| 8) Pyruvate | Total carbohydrates intake(g/d) | 0.00 [-0.11,0.12]  0.08 | -0.01 [-0.12,0.11]  0.95 | n/a | 0.00 [-0.08,0.08] |
|  | Available carbohydrates(g/d) | -0.01 [-0.12,0.10]  0.09 | 0.02 [-0.09,0.14]  0.81 | n/a | 0.01 [-0.07,0.09] |
|  | Sugar sweetened beverages and fruit juice(servings/d) | 0.01 [-0.11,0.12]  0.53 | 0.03 [-0.09,0.14]  0.82 | n/a | 0.02 [-0.06,0.10] |
|  | Fiber(g/d) | 0.05 [-0.06,0.17]  0.06 | -0.17 [-0.27, -0.05]  0.03 | n/a | -0.06 [-0.27,0.16]* |
| 9) 2-Hydroxybutyrate | Total carbohydrates intake(g/d) | -0.05 [-0.16,0.07]  0.98 | -0.09 [-0.20,0.02]  0.44 | n/a | -0.07 [-0.15,0.01] |
|  | Available carbohydrates(g/d) | -0.06 [-0.18,0.05]  0.90 | -0.11 [-0.22,0.00]  0.35 | n/a | -0.09 [-0.17,-0.01] |
|  | Sugar sweetened beverages and fruit juice(servings/d) | -0.01 [-0.13,0.10]  0.74 | -0.11 [-0.22,0.00]  0.03 | n/a | -0.06 [-0.16,0.03] |
|  | Fiber(g/d) | 0.07 [-0.05,0.18]  0.34 | 0.10 [-0.01,0.21]  0.15 | n/a | 0.08 [0.00, 0.16] |
| 10) 3-Hydroxybutyrate | Total carbohydrates intake(g/d) | -0.08 [-0.19,0.04]  0.05 | 0.06 [-0.05,0.18]  0.51 | n/a | -0.01 [-0.14,0.13] |
|  | Available carbohydrates(g/d) | -0.08 [-0.20,0.03]  0.04 | 0.07 [-0.04,0.18]  0.54 | n/a | -0.01 [-0.16,0.14]* |
|  | Sugar sweetened beverages and fruit juice(servings/d) | -0.01 [-0.13,0.10]  0.45 | 0.00 [-0.12,0.11]  0.36 | n/a | -0.01 [-0.09,0.07] |
|  | Fiber(g/d) | 0.01 [-0.11,0.12]  0.21 | -0.03 [-0.14,0.09]  0.41 | n/a | -0.01 [-0.09,0.07] |
| 11) Total hydroxybutyrate | Total carbohydrates intake(g/d) | -0.08 [-0.19,0.04]  0.12 | 0.03 [-0.08,0.14]  0.47 | -0.02 [-0.13,0.10]  0.21 | -0.02 [-0.09,0.04] |
|  | Available carbohydrates(g/d) | -0.09 [-0.20,0.03]  0.11 | 0.03 [-0.08,0.14]  0.47 | -0.02 [-0.13,0.09]  0.20 | -0.03 [-0.09,0.04] |
|  | Sugar sweetened beverages and fruit juice(servings/d) | -0.02 [-0.13,0.09]  0.45 | -0.03 [-0.14,0.08]  0.20 | 0.05 [-0.07,0.16]  0.68 | 0.00 [-0.07,0.06] |
|  | Fiber(g/d) | 0.02 [-0.09,0.13]  0.48 | 0.00 [-0.11,0.12]  0.76 | 0.00 [-0.11,0.12]  0.51 | 0.01 [-0.06,0.07] |
| 12) TMAO | Seafood (servings/d) | 0.15 [0.04, 0.26]  0.017 | 0.14 [0.03, 0.25]  0.002 | 0.08 [-0.04, 0.19]  0.12 | 0.12 [0.06, 0.19] p<.0001 |
|  | Meat(servings/d) | 0.13 [0.02,0.24]  0.05 | 0.08 [-0.04,0.19]  0.22 | 0.09 [-0.02,0.21]  0.18 | 0.10 [0.03,0.16] |
|  | Red meat(servings/d) | 0.08 [-0.03,0.20]  0.18 | 0.08 [-0.04,0.19]  0.17 | 0.10 [-0.01,0.22]  0.12 | 0.09 [0.02,0.15] |
|  | Eggs(servings/d) | 0.12 [0.01,0.23]  0.07 | 0.13 [0.02,0.24]  0.009 | 0.07 [-0.04,0.18]  0.21 | 0.11 [0.04,0.17] |
| 13) Tryptophan betaine | Nuts, seeds, and peanuts(servings/d) | 0.00 [-0.11,0.12]  0.80 | 0.25 [0.14,0.35]  <0.0001 | n/a | 0.13 [-0.11,0.35]* |
|  | Legumes(servings/d) | 0.09 [-0.02,0.20]  0.06 | -0.04 [-0.16,0.07]  0.77 | n/a | 0.02 [-0.11,0.15]* |
|  | Nuts, seeds, peanuts, and legumes(servings/d) | 0.09 [-0.03,0.2]  0.07 | 0.22 [0.11,0.33]  <0.0001 | n/a | 0.15 [0.02,0.28]* |
| 14) Tryptophan | Total protein intake(g/d) | 0.00 [-0.11,0.11]  0.88 | 0.00 [-0.12,0.11]  0.64 | -0.02 [-0.13,0.09]  0.82 | -0.01 [-0.07,0.06] |
| 15) Glycine | Total protein intake(g/d) | -0.02 [-0.13,0.10]  0.06 | -0.09 [-0.20,0.02]  0.18 | 0.07 [-0.04,0.19]  0.38 | -0.01 [-0.10, 0.08]* |

^1^Meta-analyses and associated statistical tests performed on Fisher's Z-transformed values. * indicates potentially important between-cohort heterogeneity, defined as an *I^2^* > 50.0% or P*_het_* < 0.10

## Supplementary Table 6. Results of *k*-fold cross-validation for nutrient-metabolite associations (*k*=10).

| **Food Item** | **Metabolite** | **Study** | ***R^2^*** | **SD (r^2^)** |
| --- | --- | --- | --- | --- |
| Available carbohydrates | 2-Hydroxybutyric acid | START | 0.04 | 0.05 |
|  |  | FAMILY | 0.08 | 0.12 |
|  |  | START + FAMILY | 0.03 | 0.03 |
| Citrus fruits & juice | Proline betaine | START | 0.13 | 0.07 |
|  |  | CHILD | 0.12 | 0.11 |
|  |  | FAMILY | 0.16 | 0.08 |
|  |  | START + CHILD + FAMILY | 0.05 | 0.07 |
|  |  | START + FAMILY | 0.12 | 0.06 |
| Eggs | 3-Methylhistidine | START | 0.06 | 0.05 |
|  |  | CHILD | 0.04 | 0.05 |
|  |  | FAMILY | 0.03 | 0.03 |
|  |  | START + CHILD + FAMILY | 0.05 | 0.04 |
|  |  | START + FAMILY | 0.06 | 0.04 |
| Fruit | Hippuric acid | START | 0.05 | 0.04 |
|  |  | CHILD | 0.04 | 0.04 |
|  |  | FAMILY | 0.09 | 0.09 |
|  |  | START + CHILD + FAMILY | 0.03 | 0.03 |
|  |  | START + FAMILY | 0.04 | 0.04 |
| Nuts, seeds, peanuts, and legumes | Tryptophan betaine | START | 0.04 | 0.03 |
|  |  | FAMILY | 0.1 | 0.08 |
|  |  | START + FAMILY | 0.05 | 0.03 |
| Red meat | Carnitine | START | 0.04 | 0.04 |
|  |  | CHILD | 0.04 | 0.05 |
|  |  | FAMILY | 0.05 | 0.09 |
|  |  | START + CHILD + FAMILY | 0.06 | 0.03 |
|  |  | START + FAMILY | 0.03 | 0.04 |
| Seafood | TMAO | START | 0.08 | 0.12 |
|  |  | CHILD | 0.05 | 0.03 |
|  |  | FAMILY | 0.06 | 0.05 |
|  |  | START + CHILD + FAMILY | 0.08 | 0.04 |
|  |  | START + FAMILY | 0.09 | 0.06 |

## Supplementary Table 7. Meta-regression summary for serum metabolite-food pairs that exhibited heterogeneity of effects upon meta-analysis

| **Metabolite** | **Food group/item** | **Non-fasting**  **(summary *r* estimate)^1^** | **Fasting**  **(summary *r* estimate)^1^** | **Q statistic,**  **P value^2^** |
| --- | --- | --- | --- | --- |
| Proline betaine | Citrus fruits | 0.36 (0.26, 0.46) | 0.25 (0.18, 0.33) | 2.98, P=0.08* |
| Proline betaine | Citrus fruits & juice | 0.41 (0.31, 0.5) | 0.43 (0.36, 0.49) | 0.10, P=0.75 |
| Proline betaine | Citrus juice | 0.24 (0.09, 0.37) | 0.41 (0.32, 0.5) | 4.27, P=0.04* |
| 3-Methylhistidine | Eggs | 0.15 (0.03, 0.27) | 0.19 (0.11, 0.27) | 0.30, P=0.58 |
| 3-Methylhistidine | Chicken | 0.28 (0.03, 0.5) | 0.25 (0.07, 0.41) | 0.04, P=0.85 |
| 3-Methylhistidine | Total protein intake | 0.14 (0.02, 0.25) | 0.06 (-0.02, 0.14) | 1.28, P=0.26 |
| Carnitine | Eggs | 0.08 (-0.03, 0.19) | 0.06 (-0.02, 0.14) | 0.06, P=0.81 |
| Carnitine | Red meat | 0.08 (-0.04, 0.19) | 0.1 (0.02, 0.18) | 0.06, P=0.80 |
| Hippuric acid | Fruit | 0.13 (-0.04, 0.29) | 0.2 (0.08, 0.31) | 0.49, P=0.49 |
| Hippuric acid | Vegetables | 0.19 (-0.07, 0.43) | 0.14 (-0.05, 0.32) | 0.10, P=0.75 |
| Total Hydroxybutyric acid | Total carbohydrates intake | -0.07 (-0.18, 0.04) | -0.07 (-0.15, 0.01) | 0.01, P=0.92 |
| Total Hydroxybutyric acid | Available carbohydrates | -0.07 (-0.19, 0.04) | -0.07 (-0.15, 0.01) | 0.01, P=0.93 |
| TMAO | Eggs | 0.07 (-0.04, 0.18) | 0.13 (0.05, 0.2) | 0.60, P=0.44 |
| TMAO | Meat | 0.09 (-0.02, 0.2) | 0.1 (0.02, 0.18) | 0.03, P=0.86 |
| TMAO | Red meat | 0.1 (-0.01, 0.21) | 0.08 (0, 0.16) | 0.10, P=0.75 |
| TMAO | Seafood | 0.08 (-0.04, 0.19) | 0.15 (0.07, 0.22) | 0.95, P=0.33 |

^1^Meta-analyses and associated statistical tests performed on Fisher's Z-transformed values. Table presents back-transformed values.

^2^Presents Q-statistic, and P-value for heterogeneity between fasting and non-fasting samples; * indicates potentially important between-cohort heterogeneity, defined as P*_het_* < 0.10

# Supplementary Figures

## Supplementary Figure 1. Flowchart of study participant selection for metabolomic analyses from three ongoing birth cohorts

**CONSORT 2010 Flow Diagram**

**START** Analysed (n=300)

- 100 High DQS
- 100 Medium DQS
- 100 High DQS

**FAMILY** Analysed (n=300)

- 100 High DQS
- 100 Medium DQS
- 100 High DQS

**CHILD** Analysed (n=300)

- 100 High DQS
- 100 Medium DQS
- 100 High DQS

**Chemical Analysis**

**CHILD** (n=300/3,296)

- 100 from top 10^th^ percentile of DQS
- 100 from bottom 10^th^ percentile of DQS
- 100 from remaining 3,096

**FAMILY**  (n=300/816)

- 100 top-ranked DQS
- 100 bottom-ranked DQS
- 100 selected at random from remaining 616

**START** (n=300/1,012)

- 100 from top 10^th^ percentile of DQS
- 100 from bottom 10^th^ percentile of DQS
- 100 from remaining 812

**Enrollment**

Assessed for eligibility (n=5,124)

(1,012 from START + 816 from FAMILY + 3,296 from CHILD)

*Enrolled prior to January 1, 2018*

Excluded (n=123)

- Did not provide serum sample and complete, plausible FFQ (n=123)

**Selection**

Eligible for selection (n=5,001)

Supplementary Figure 2. Histograms of diet quality score. Left column, “Diet quality score (DQS)”, shows the distribution of untransformed, raw scores; right column, “Log(Capped DQS)”, shows the natural-logarithm-transformed winsorized scores used in all analyses.

**
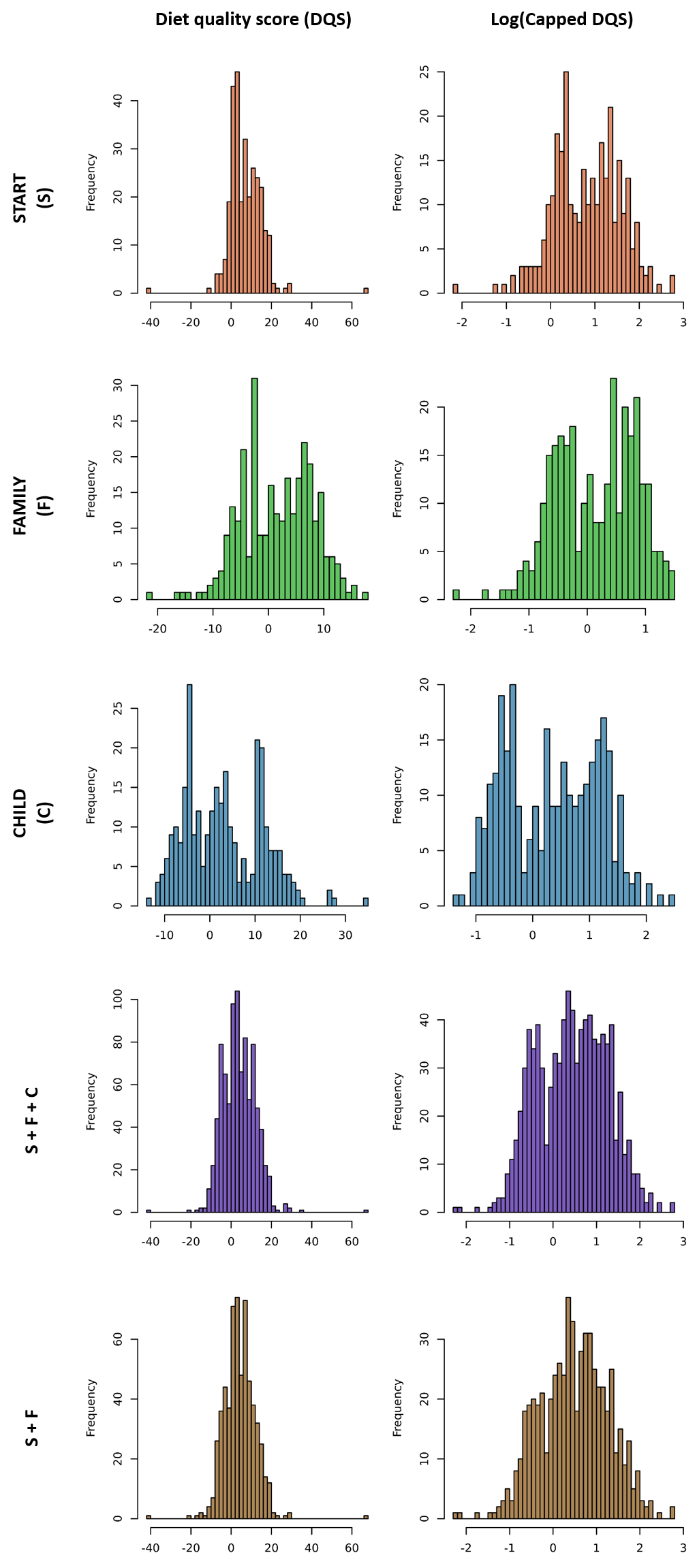
**

Supplementary Figure 3. Histograms of healthy diet score. Left column, “Healthy diet score (HDS)”, shows the distribution of untransformed, raw scores; right column, “Log(Capped HDS)”, shows the natural-logarithm-transformed winsorized scores used in all analyses.

**
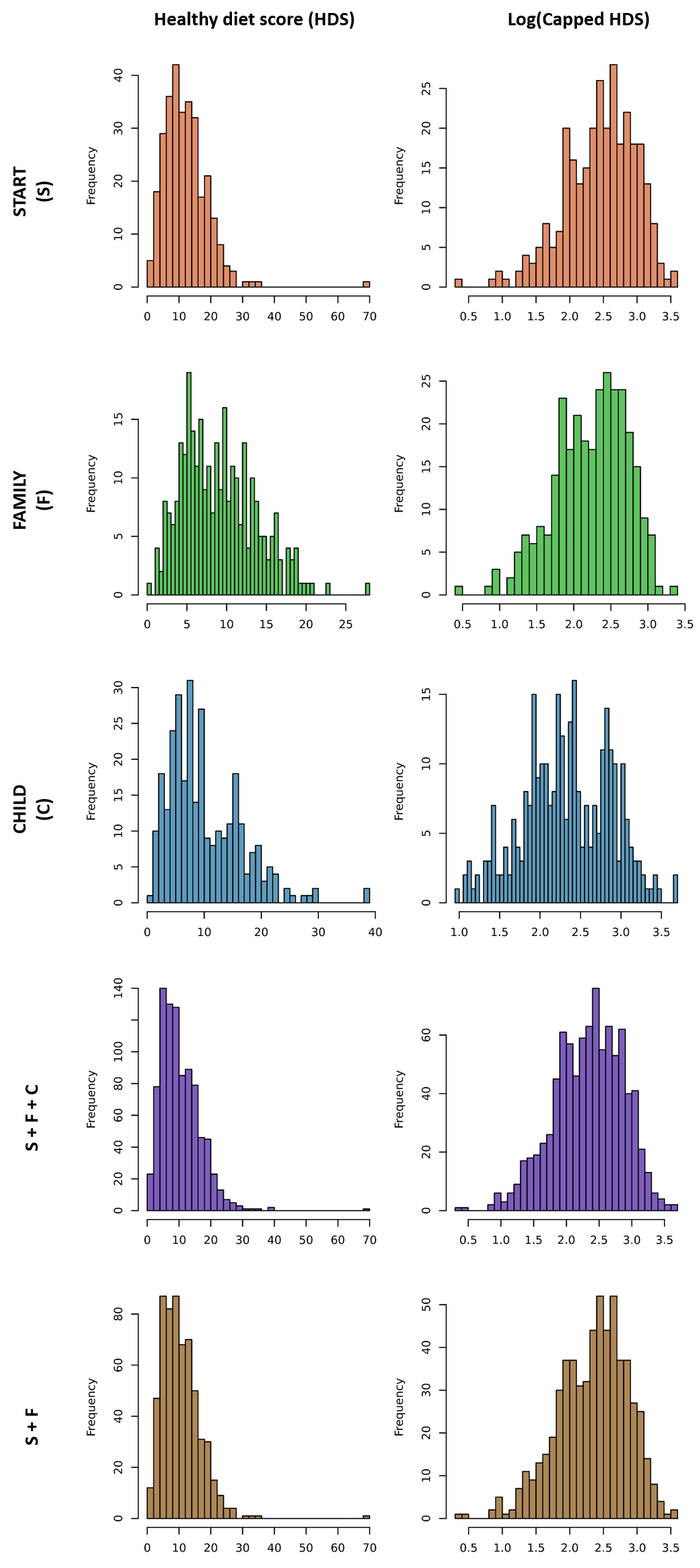
**

Supplementary Figure 4. Histograms of unhealthy diet score. Left column, “Unhealthy diet score (UDS)”, shows the distribution of untransformed, raw scores; right column, “Log(Capped UDS)”, shows the natural-logarithm-transformed winsorized scores used in all analyses.

**
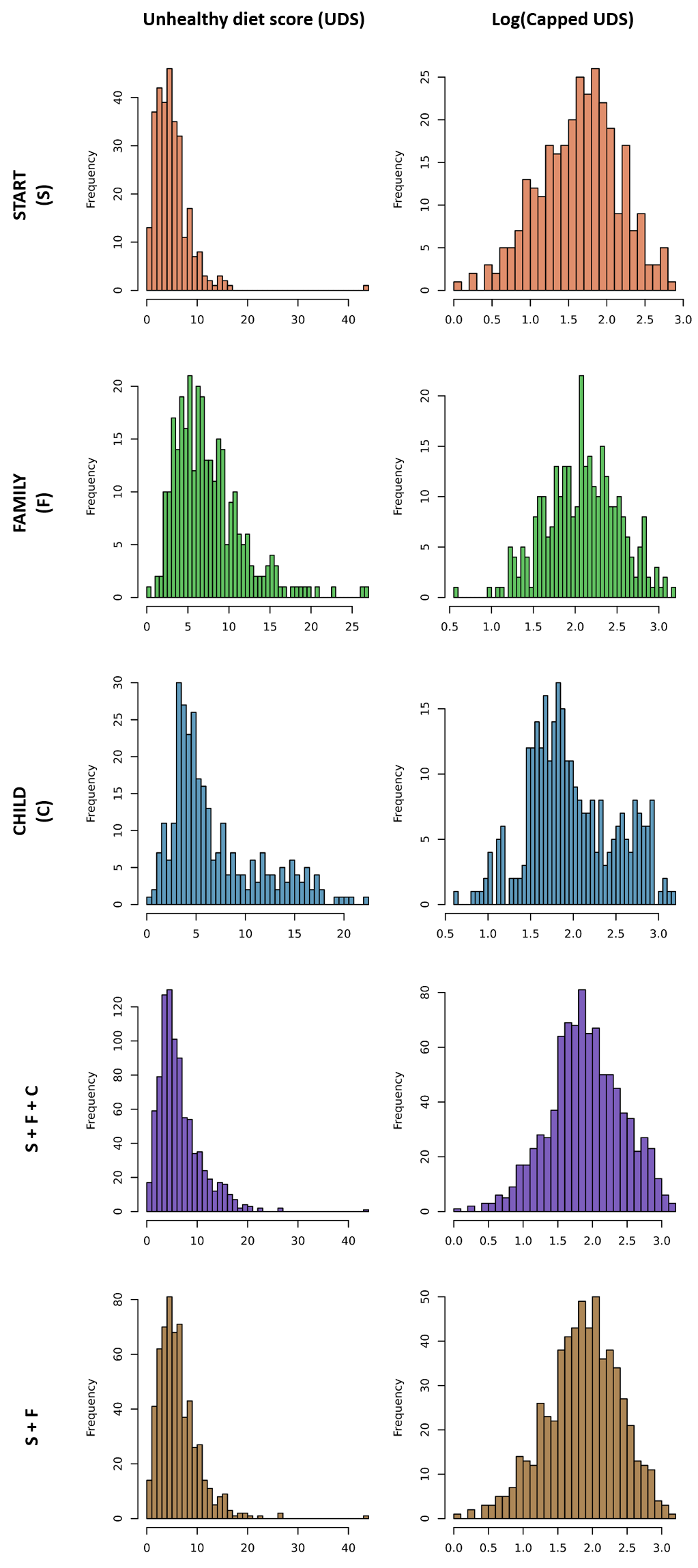
**

Supplementary Figure 5. Box plots of healthy diet sub score (A), unhealthy diet sub score (B), and diet quality score (C) by cohort.


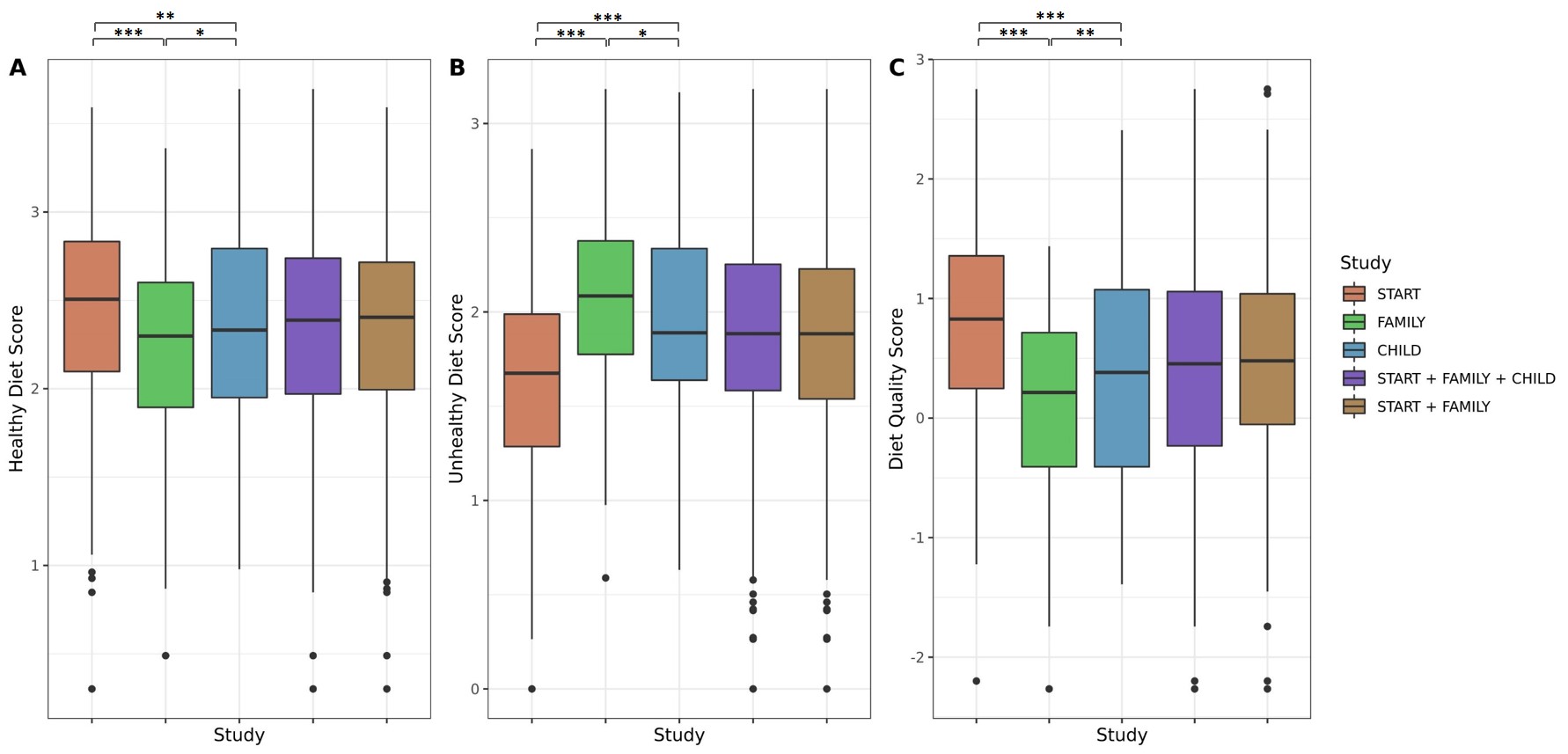


*** P-value <0.001 from ANOVA test; ** 0.01 < P -value ≤ 0.001 from ANOVA test; * 0.05 < P-value ≤ 0.01 from ANOVA test.

Supplementary Figure 6 a-c. Volcano plot of results of t-test screen of mean serum metabolite responses versus high & low diet quality for three maternal birth cohorts. START, **A**; FAMILY, **B**; and CHILD, **C;**

### Supplementary Figure 6a. START

### Supplementary Figure 6b. FAMILY

### Supplementary Figure 6c. CHILD

The y-axis displays (-log10)-transformed p-values. The higher the value the smaller the p-value. A value of 1 equates to p=0.10 threshold. The x-axis displays fold-changes of metabolite means comparing high/low diet quality. Values are log_2_ transformed so that distances in negative and positive directions are equidistant from no-change (=0) vertical axis. Points further to the right denote higher metabolite concentration in those with a high diet quality score compared to those with a low diet quality score.

Supplementary Figure 7. Association of TMAO with fish and seafood intake by cohort.


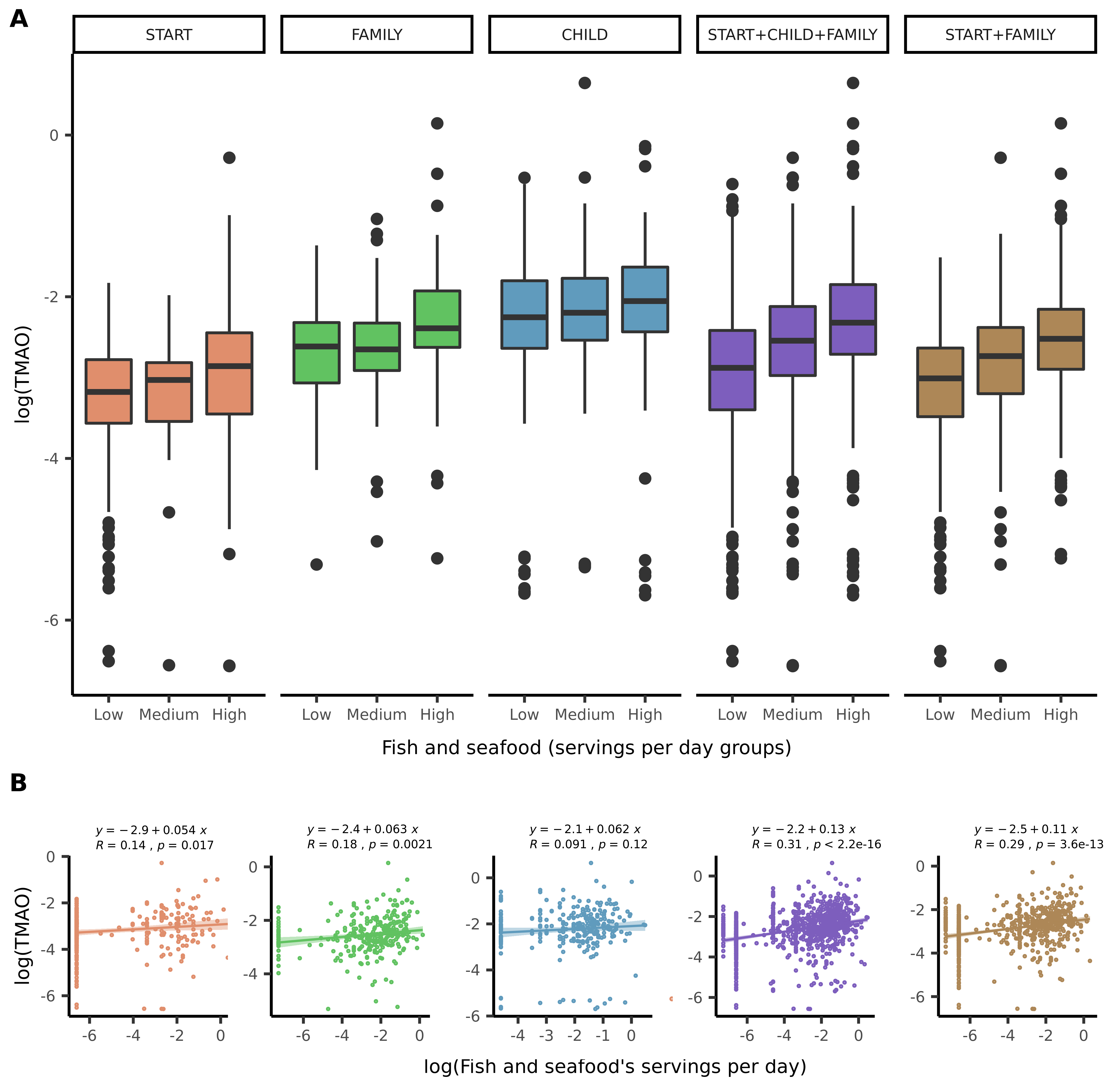


Top panel (A) shows boxplots of serum TMAO relative peak area vs. self-reported fish and seafood within low, medium, and high intake (servings/d) as terciles; bottom panel (B) shows scatterplots of serum TMAO relative peak area vs. log-transformed servings/d of fish and seafood. Slope of regression line in panel B represented as *log (metabolite)= b + m(# servings/d)*; R = Pearson’s correlation coefficient; P = p-value for association.

Supplementary Figure 8. Association of carnitine with red meat intake by cohort.


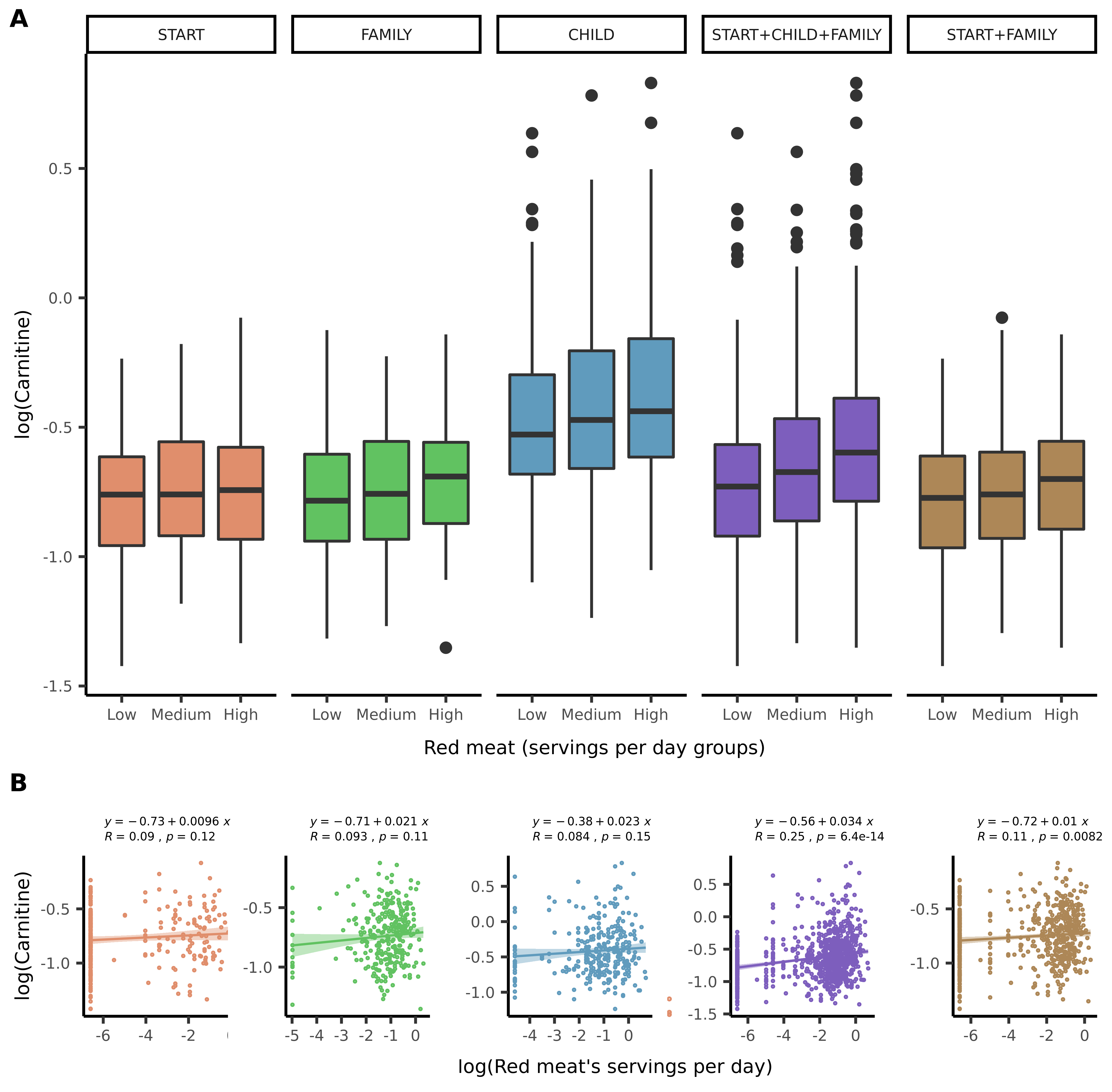


Top panel (A) shows boxplots of serum carnitine relative peak area vs. self-reported red meat within low, medium, and high intake (servings/d) as terciles; bottom panel (B) shows scatterplots of serum carnitine relative peak area vs. log-transformed servings/d of red meat. Slope of regression line in panel B represented as *log (metabolite)= b + m(# servings/d)*; R = Pearson’s correlation coefficient; P = p-value for association.

Supplementary Figure 9. Association of hippuric acid with fruit intake by cohort.


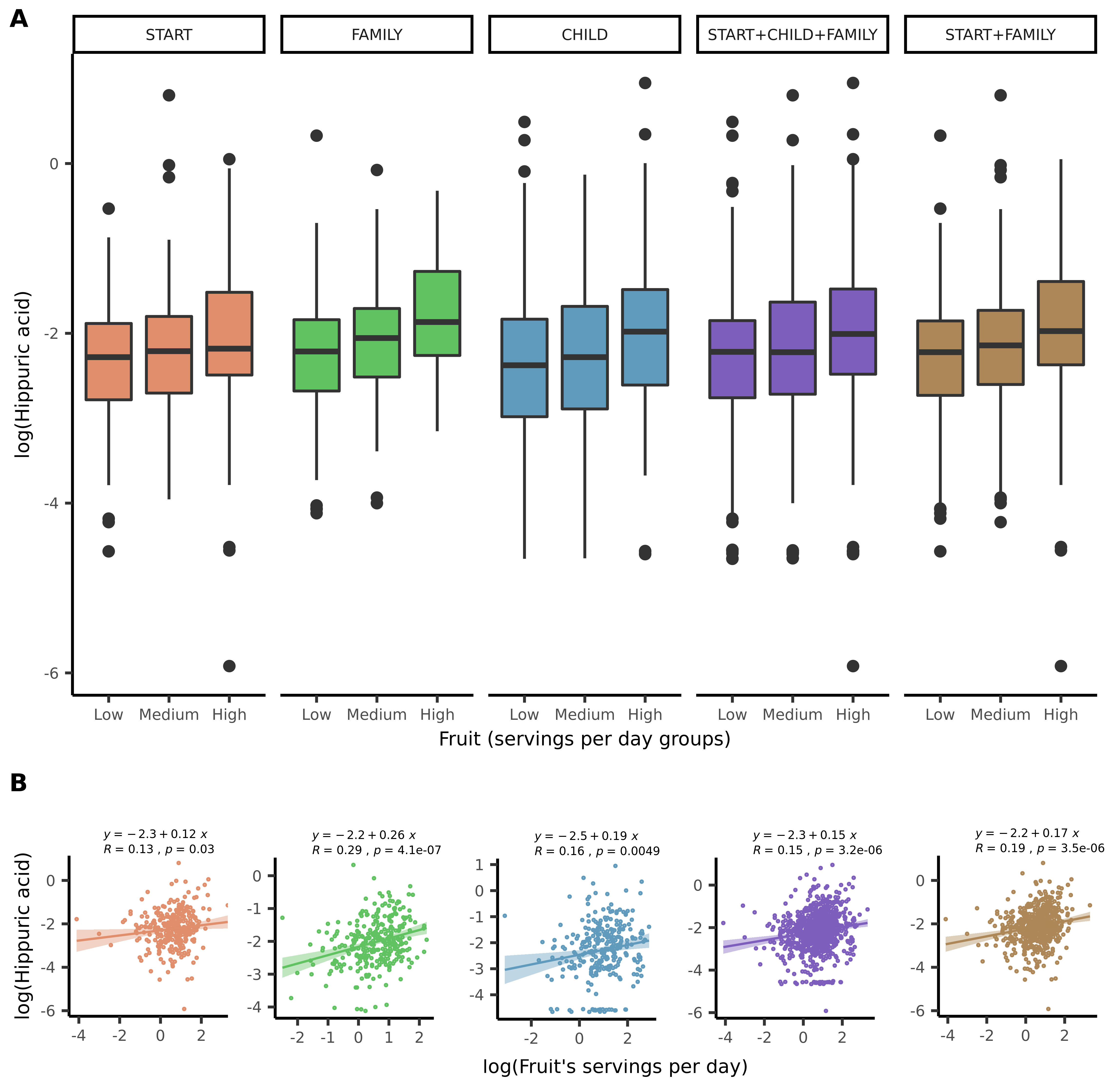


Top panel (A) shows boxplots of serum hippuric acid relative peak area vs. self-reported fruit within low, medium, and high intake (servings/d) as terciles; bottom panel (B) shows scatterplots of serum hippuric acid relative peak area vs. log-transformed servings/d of fruit. Slope of regression line in panel B represented as *log (metabolite)= b + m(# servings/d)*; R = Pearson’s correlation coefficient; P = p-value for association.

Supplementary Figure 10. Association of tryptophan betaine with nuts and legume intake by cohort.


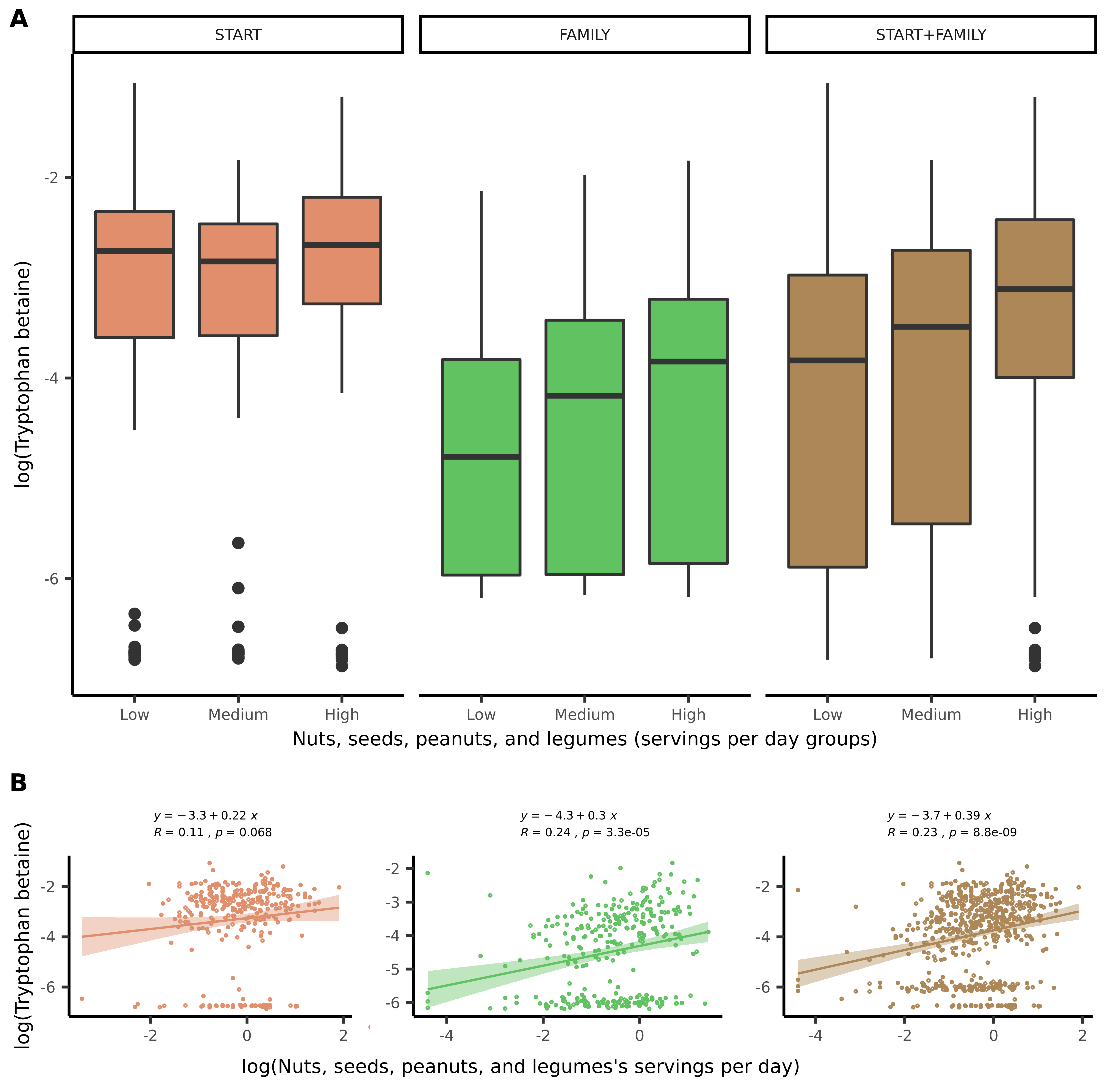


Top panel (A) shows boxplots of serum tryptophan betaine relative peak area vs. self-reported seeds, peanuts, and legumes within low, medium, and high intake (servings/d) as terciles; bottom panel (B) shows scatterplots of serum tryptophan betaine relative peak area vs. log-transformed servings/d of seeds, peanuts, and legumes. Slope of regression line in panel B represented as *log (metabolite)= b + m(# servings/d)*; R = Pearson’s correlation coefficient; P = p-value for association. *Tryptophan betaine was not detected with adequate frequency and precision in serum samples collected from the CHILD cohort.*

Supplementary Figure 11. Association of 3-methylhistidine with egg intake by cohort.

**
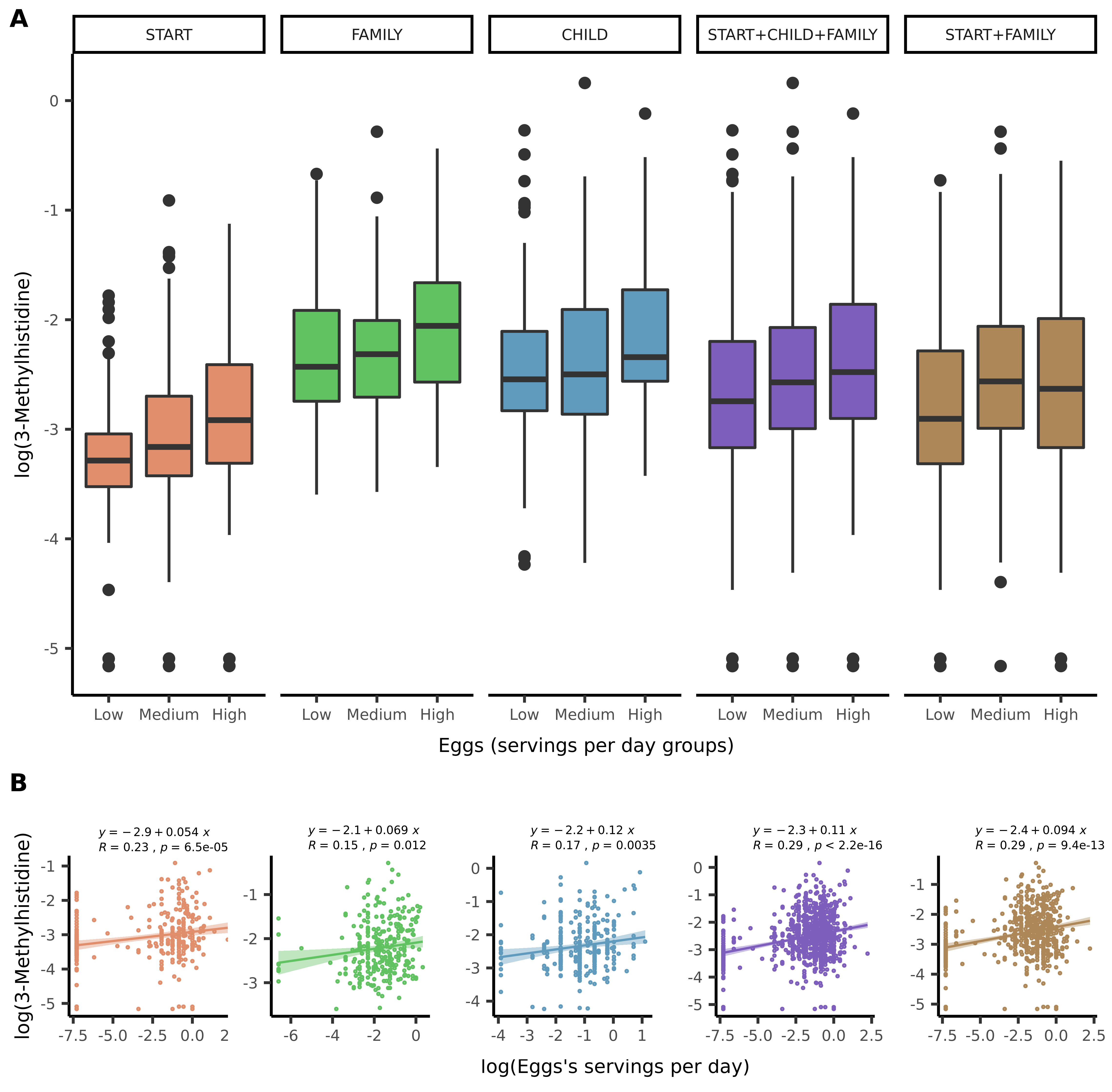
**

Top panel (A) shows boxplots of serum 3-methylhistidine relative peak area vs. self-reported egg intake within low, medium, and high intake (servings/d) as terciles; bottom panel (B) shows scatterplots of serum 3-methylhistidine relative peak area vs. log-transformed servings/d of eggs. Slope of regression line in panel B represented as *log (metabolite)= b + m(# servings/d)*; R = Pearson’s correlation coefficient; P = p-value for association.

# REFERENCES

1. Morrison KM, Anand SS, Yusuf S, Atkinson SA, Schulze KM, Rao-Melacini P, McQueen MJ, McDonald S, Persadie R, Hunter B, et al. Maternal and pregnancy related predictors of cardiometabolic traits in newborns. PLoS One. 2013;8(2):e55815. Epub 2013/02/19. doi: 10.1371/journal.pone.0055815. PubMed PMID: 23418462; PubMed Central PMCID: PMCPMC3572188.

2. Morrison KM, Atkinson SA, Yusuf S, Bourgeois J, McDonald S, McQueen MJ, Persadie R, Hunter B, Pogue J, Teo K, et al. The Family Atherosclerosis Monitoring In earLY life (FAMILY) study: rationale, design, and baseline data of a study examining the early determinants of atherosclerosis. Am Heart J. 2009;158(4):533-9. Epub 2009/09/29. doi: 10.1016/j.ahj.2009.07.005. PubMed PMID: 19781411.

3. Subbarao P, Anand SS, Becker AB, Befus AD, Brauer M, Brook JR, Denburg JA, HayGlass KT, Kobor MS, Kollmann TR, et al. The Canadian Healthy Infant Longitudinal Development (CHILD) Study: examining developmental origins of allergy and asthma. Thorax. 2015;70(10):998-1000. doi: 10.1136/thoraxjnl-2015-207246.

4. Tamana SK, Ezeugwu V, Chikuma J, Lefebvre DL, Azad MB, Moraes TJ, Subbarao P, Becker AB, Turvey SE, Sears MR, et al. Screen-time is associated with inattention problems in preschoolers: Results from the CHILD birth cohort study. PLoS One. 2019;14(4):e0213995. Epub 2019/04/18. doi: 10.1371/journal.pone.0213995. PubMed PMID: 30995220; PubMed Central PMCID: PMCPMC6469768.

5. Anand SS, Vasudevan A, Gupta M, Morrison K, Kurpad A, Teo KK, Srinivasan K, Investigators SCS. Rationale and design of South Asian Birth Cohort (START): a Canada-India collaborative study. BMC Public Health. 2013;13:79. Epub 2013/01/30. doi: 10.1186/1471-2458-13-79. PubMed PMID: 23356884; PubMed Central PMCID: PMCPMC3585827.

6. Kuehnbaum NL, Kormendi A, Britz-McKibbin P. Multisegment injection-capillary electrophoresis-mass spectrometry: a high-throughput platform for metabolomics with high data fidelity. Anal Chem. 2013;85(22):10664-9. Epub 2013/11/08. doi: 10.1021/ac403171u. PubMed PMID: 24195601.

7. DiBattista A, McIntosh N, Lamoureux M, Al-Dirbashi OY, Chakraborty P, Britz-McKibbin P. Temporal Signal Pattern Recognition in Mass Spectrometry: A Method for Rapid Identification and Accurate Quantification of Biomarkers for Inborn Errors of Metabolism with Quality Assurance. Anal Chem. 2017;89(15):8112-21. Epub 2017/06/27. doi: 10.1021/acs.analchem.7b01727. PubMed PMID: 28648083.

8. Wild J, Shanmuganathan M, Hayashi M, Potter M, Britz-McKibbin P. Metabolomics for improved treatment monitoring of phenylketonuria: urinary biomarkers for non-invasive assessment of dietary adherence and nutritional deficiencies. Analyst. 2019;144(22):6595-608. Epub 2019/10/15. doi: 10.1039/c9an01642b. PubMed PMID: 31608347.

9. Shanmuganathan M, Kroezen Z, Gill B, Azab S, de Souza RJ, Teo K, Desai D, Befus AD, Morrison K, Atkinson SA, et al. The Maternal Serum Metabolome by Multisegment Injection-Capillary Electrophoresis-Mass Spectrometry: A High Throughput Platform for Large-scale Epidemiological Studies. Nature Protocols (accepted), 2020. Sept. 24, 2020.
